# Functional and behavioral outcomes in pediatric adrenal carcinoma under mitotane therapy: a caregiver-reported pilot study

**DOI:** 10.1530/EO-26-0010

**Published:** 2026-06-05

**Authors:** Heidi Frey, Maria Riedmeier, Johanna Krüger, Carl Friedrich Classen, Nerea Domínguez-Pinilla, Maria Fragoso, Steffen Fuchs, Dominika Janus, Mouna Mezoued, Andrea Mocna, Jessica Munarin, Soraya Puglisi, Hilal Susam Sen, Gerdi Tuli, Justyna Walenciak, Bilgehan Yalcin, Martin Fassnacht, Verena Wiegering

**Affiliations:** ^1^ University Hospital Würzburg, Department of Pediatrics, Division of Pediatric Hematology, Oncology and Stem Cell Transplantation, University of Wuerzburg, Wuerzburg, Germany; ^2^ Pediatric Hematology and Oncology, Goethe-University Frankfurt, Frankfurt, Germany; ^3^ Division of Pediatric Oncology, Hematology and Palliative Medicine Section, Department of Pediatrics and Adolescent Medicine, University Medicine Rostock, Rostock, Germany; ^4^ Pediatric Hematology and Oncology Unit, University Hospital 12 de Octubre, I+12 Research Institute, Madrid, Spain; ^5^ Department of Pediatrics, University of São Paulo, São Paulo, Brazil; ^6^ Department of Pediatric Oncology and Hematology, Charité – Universitätsmedizin Berlin, Berlin, Germany; ^7^ Berlin Institute of Health at Charité – Universitätsmedizin Berlin, Berlin, Germany; ^8^ German Cancer Consortium (DKTK), Partner Site Berlin, A Partnership Between DKFZ and Charité-Universitätsmedizin Berlin, Berlin, Germany; ^9^ Department of Pediatric and Adolescent Endocrinology, Jagiellonian University Medical College, University Children Hospital, Krakow, Poland; ^10^ Department of Endocrinology and Metabolism Bologhine Hospital, Algiers, Algeria; ^11^ Research Laboratory of Endocrinology and Metabolism (LEM1), Benyoucef Benkhedda University, Algiers, Algeria; ^12^ Department of Pediatric Hematology and Oncology, Comenius University Faculty of Medicine and National Institute of Children’s Diseases, Bratislava, Slovakia; ^13^ Department of Public Health and Pediatrics, University of Turin, Turin, Italy; ^14^ Internal Medicine, Department of Clinical and Biological Sciences, S. Luigi Gonzaga Hospital, University of Turin, Orbassano, Italy; ^15^ Department of Pediatric Oncology, Hacettepe University Faculty of Medicine, Ankara, Turkey; ^16^ Department of Pediatric Endocrinology, Regina Margherita Children’s Hospital, Turin, Italy; ^17^ Department of Pediatrics, University of Turin, Turin, Italy; ^18^ Department of Pediatrics, Oncology and Hematology, Medical University of Lodz, Lodz, Poland; ^19^ Department of Medicine, Division of Endocrinology and Diabetes, University Hospital, University of Wuerzburg, Wuerzburg, Germany; ^20^ Comprehensive Cancer Centre CCC WERA, University of Wuerzburg Medical Centre, Wuerzburg, Germany

**Keywords:** pediatric adrenocortical carcinoma, pediatric adrenocortical tumor, mitotane, adverse side effects, neuropsychological long-term outcome

## Abstract

**Objective:**

Mitotane is the standard therapy for advanced pediatric adrenocortical carcinoma (pACC). With improving survival, treatment-related long-term side effects and their impact on everyday functioning are becoming increasingly relevant. This multicenter pilot study provides first insights into this underexplored topic and lays the groundwork for future prospective investigations.

**Design and methods:**

Caregivers of children with pACC treated with mitotane completed an online questionnaire. Based on these retrospective caregiver-reported data, treatment-related side effects and growth parameters were assessed. Using two validated instruments – Child and Adolescent Scale of Participation (CASP) and Strengths and Difficulties Questionnaire (SDQ) – caregivers rated functional participation and behavioral outcomes. We classified patients according to ongoing versus completed mitotane therapy and compared them with a sibling control group.

**Results:**

The cohort included 24 children with pACC from eight countries, predominantly with advanced tumors (stage III–IV: 66.7%, median age: 7.4 years, 41.7% females). Half had completed mitotane therapy, and half were still receiving it. Most patients experienced relevant side effects, with adrenal insufficiency being the most frequent ongoing limitation. Patients on ongoing mitotane therapy showed reduced functional participation and more pronounced behavioral difficulties, whereas those who had completed therapy had outcomes comparable to their siblings.

**Conclusion:**

The findings of this pilot study suggest temporary impairments in functional participation and psychosocial behavior during mitotane treatment, with improvement after therapy completion. In contrast, endocrine sequelae, particularly adrenal insufficiency, may persist beyond treatment. These observations underscore the need for standardized long-term endocrine follow-up and support the feasibility of larger, prospective studies to evaluate long-term neuropsychological outcome, especially in younger children.

## Introduction

Adrenocortical carcinoma in pediatric patients (pACC) is an extremely rare, aggressive endocrine malignancy of the adrenal cortex, with an incidence of 0.2–0.3 cases per million children under the age of 20 per year (1, 2, 3). With a 5-year survival rate between 30 and 70%, depending on the stage at presentation, pACC has an unfavorable prognosis. It is frequently associated with genetic predisposition syndromes, such as Li–Fraumeni and Beckwith–Wiedemann syndromes (4, 5). In contrast to adult ACC, the vast majority of pediatric tumors are hormone-producing, most commonly with androgen or cortisol excess. Patients may present with virilization, Cushing’s syndrome, or mixed endocrine symptoms (1, 4, 6). While complete *en bloc* surgical resection represents the primary and most effective therapy, a multimodal treatment, including chemotherapy, radiotherapy, and the adrenolytic agent mitotane, is typically required, especially in cases of advanced disease or incomplete resection (7, 8, 9). Despite its narrow therapeutic window and broad, heterogeneous toxicity profile, including neurotoxic effects, mitotane is the only substance with specific adrenolytic activity on the adrenal cortex (7, 10, 11, 12). Therefore, mitotane remains a cornerstone of treatment with the potential to improve prognosis and extend overall survival (13, 14, 15).

As therapeutic strategies for pACC continue to advance and survival rates gradually improve, the relevance of understanding long-term adverse effects becomes increasingly important. Although neurological toxicity is known to correlate with elevated mitotane plasma levels and recovery after termination of treatment has been reported (12, 13), the current literature provides almost no evidence regarding psychosocial outcomes or functional participation in this population. This is particularly relevant for pediatric patients, whose treatment coincides with sensitive periods of brain development, raising concern that mitotane-related neuropsychological effects may have lasting consequences for daily participation.

To begin addressing this understudied topic in children with pACC, we conducted a multicenter, caregiver-reported online survey. This pilot study aimed to generate preliminary insights into mitotane-related long-term effects on functional participation and psychosocial behavior in children with pACC and to evaluate the feasibility of future larger prospective studies.

## Methods

### Study design and data collection

This multicenter pilot study was observational, exploratory, and retrospective in design, using caregiver-reported data. It received approval from the Würzburg local ethics committee (20231016 20102, Würzburg, Germany 2023) and was available to the patients’ respective treating physicians. We initially sent a total of 52 QR codes linking to an online questionnaire via email to the physicians, who then forwarded them to families of children diagnosed with pediatric adrenocortical carcinoma. A total of 24 caregivers eventually completed the survey. The participation was voluntary, and all data were transferred anonymously. In addition to a histopathologically confirmed diagnosis of pACC, independent of relapse or active disease status, inclusion criteria for participation were ongoing or completed mitotane therapy and age below 21 years at the time of diagnosis. The questionnaire was available online between July 2024 and October 2025. We include both patients on ongoing mitotane and those who had completed treatment to ensure sufficient sample size and allow comparison of effects during and after therapy. In addition, we included sixteen siblings as a control group due to shared family and genetic factors. To improve comparability, caregivers were instructed to complete the questionnaire for the sibling closest in age to the patient if more than one sibling was available. We designed the questionnaire via the online platform https://evasys.de/. The original English version was translated into seven different languages using OpenAI ChatGPT, after which native speakers reviewed the translations to ensure content accuracy and accessibility for all participants. The supplementary file comprises the complete questionnaire and three distinct representations of the response data. The material was fully provided by evasys and constitutes the source for all tables and figures in the manuscript.

### Auxological assessment: BMI and length Z-scores

To obtain an approximate overview of patients’ auxological status, caregivers reported height and weight at diagnosis and at the time of the survey. Based on these self-reported data, body mass index (BMI; weight in kg/height in m^2^) was calculated, and age- and sex-adjusted Z-scores were derived to ensure comparability across patients. We used the LMS method according to Cole *et al.* (16), applying the following formula:

$$Z=\frac{{\left(\frac{X}{M}\right)}^{L}-1}{L\times S}$$

where *X* is the measured value (BMI or length), *M* is the population mean, *L* is the Box–Cox power that normalizes skewed data, and *S* is the coefficient of variation (17). WHO LMS reference data from the 2006 standards (for <5 years old) and the 2007 reference (≥5 years) were used (18, 19, 20). Age at survey was approximated to the nearest year based on the interval since diagnosis. Standard WHO cutoffs were applied to classify BMI Z-scores in underweight (<−2), mild underweight (−1 to −2), normal weight (−1 to +1), overweight (+1 to +2), and obesity (>+2) and length Z-scores in stunting (<−2), normal stature (−2 to +2), and tall stature (>+2) (21, 22).

### Functional participation: CASP score

To assess the functional and social participation in daily life, the internationally validated Child and Adolescent Scale of Participation (CASP) by Gary Bedell was applied (23). Caregivers rated their child’s participation in 20 items across four domains: home, school, community, and daily living activities. Each item was scored on a 4-point scale (1 = age-expected to 4 = unable), while ‘not applicable’ responses were excluded from analysis (23, 24). The CASP scores, which range from 0 to 100%, can be classified into four levels of participation (100–97.5 = full participation, 97.5–81.0 = somewhat limited, 81.0–68.5 = limited, and ≤68.5 = very limited) (25). The overall and domain-specific CASP scores were calculated for all participants using the formula: (sum of items/(number of items × 4)) × 100. To investigate potential differences in participation, results were compared across three groups: patients currently receiving mitotane, patients who had completed mitotane therapy, and a combined group of siblings, regardless of whether the corresponding patient was receiving ongoing or completed therapy.

To evaluate a potential influence of parental educational background on patients’ participation scores, parental education was categorized into low (no formal school certificate), medium (upper secondary/middle school), and high education levels (university entrance qualifications) based on the highest completed school qualification of one parent (26).

### Behavioral outcome: SD-questionnaire

To assess emotional and behavioral problems, the Strengths and Difficulties Questionnaire (SDQ) – originally developed by Robert Goodman (1997) – was administered (27, 28). The number of items was deliberately reduced to nine questions, to minimize survey fatigue and increase the likelihood of completion. One to two items were chosen from each of the five SDQ subscales. Each item is rated on a three-point scale (1 = not true to 3 = certainly true).

If the patients had siblings, the final two sections of the questionnaire contained the same CASP and SDQ items but referred to the siblings.

### Statistical methods

Statistical analyses and graphical visualizations were performed using GraphPad Prism, version 10. Tables were created in Microsoft Excel, and the side-effect graphic was exported from the evasys survey platform. Categorical variables were compared using Fisher’s exact test, while continuous non-normally distributed variables, such as patient characteristics between the mitotane ongoing and completed groups, were compared using the Mann–Whitney U test. Because most variables were non-normally distributed, non-parametric tests were applied. Group comparisons across more than two independent groups used the Kruskal–Wallis test with Dunn’s post hoc analysis. Comparisons of CASP scores across domains were analyzed using the Friedman test. The Wilcoxon matched-pairs test was used for comparison of patient–sibling pairs. Free-text responses were evaluated using simple content analysis by grouping answers into thematic categories. A *P*-value <0.05 was considered statistically significant.

## Results

### Patient characteristics

A total of 24 pediatric patients diagnosed with pACC between 1995 and 2024 were included. The mean age at diagnosis was 7.4 years (=88.5 months; 95% CI: 59.8–117.4). Patients originated from eight different countries, with a slight male predominance (*n* = 10; 58.3%). At diagnosis, most tumors were in advanced stages (stage III–IV: *n* = 16; 66.7%). All but one patient underwent surgical tumor resection, and all received mitotane therapy. Cytotoxic chemotherapy was administered in 62.5% (*n* = 15), and radiotherapy was administered in 20.8% (*n* = 5). At the time of data collection, 50% were still on mitotane treatment. The mean duration of mitotane treatment for the whole cohort was 17.7 months (range: 3–46; 95% CI: 12.9–22.5), and it was slightly longer among patients who had already completed therapy at 18.5 months (min/max: 3/46; 95% CI: 12.4–24.6). Clinical remission was achieved in most cases (*n* = 16; 66.7%). Pre-existing illnesses as risk factors for pACC were infrequent.

In the completed-therapy group, the mean time since mitotane discontinuation was 6.9 years (range: 0–27 years). Six patients had completed therapy less than 5 years prior, and five had completed therapy more than 5 years prior. Data on time since treatment discontinuation were missing for one patient.

When comparing the two subgroups, mitotane ongoing and completed, three statistically significant differences were observed. As expected, treatment duration differed, with patients still on mitotane having a shorter mean duration (8.2 months, 95% CI: −1.7–18.1) compared with those who had completed therapy (18.5 months, 95% CI: 12.4–24.6; *P* = 0.0042**). Treatment modalities also differed, with more patients in the ongoing group received chemotherapy (11 vs 4; *P* = 0.094**) and radiotherapy (5 vs 0; *P* = 0.037*). No other patient characteristics showed statistically significant differences between the groups (see Table 1).

**Table 1:** Cohort characteristics, including age at diagnosis, sex, country, tumor stage, tumor treatment, current mitotane therapy, duration of mitotane therapy, remission status, and pre-existing illnesses, stratified by mitotane ongoing and mitotane completed group (*n* = 12 each); *n* = number of patients.

Cohort characteristics		All patients	Mitotane ongoing^a^	Mitotane completed^a^
		*n* = 24	*n* = 12	*n* = 12
Age at diagnosis (*n*; %)	Median/mean/range (months)	88.5/69.5/1–216	74.5/95.9/1–198	60/81.2/1.2–216
	<4 years	7; 29.2	4; 33.3	3; 25.0
	4–11.9 years	11; 45.8	4; 33.3	7; 58.3
	12–18 years	6; 25.0	4; 33.3	2; 16.7
Sex (*n*; %)	Female	10; 41.7	4; 33.3	6; 50.0
	Male	14; 58.3	8; 66.7	6; 50.0
Country (*n*; %)	Germany	6; 25.0	2; 16.7	4; 33.3
	Italy	4; 16.7	2; 16.7	2; 16.7
	Algeria	3; 12.5	3; 25.0	0; 0.0
	Poland	2; 8.3	2; 16.7	0; 0.0
	Spain	2; 8.3	1; 8.3	1; 8.3
	Brazil	2; 8.3	0; 0.0	2; 16.7
	Slovakia	1; 4.2	1; 8.3	0; 0.0
	Turkey	4; 16.7	3; 25.0	1; 8.3
Tumor stage (*n*; %)	I	1; 4.2	1; 8.3	0; 0.0
	II	3; 12.5	1; 8.3	2; 16.7
	III	6; 25.0	4; 33.3	2; 16.7
	IV	10; 41.7	4; 33.3	6; 50.0
	Unknown	4; 16.7	2; 16.7	2; 16.7
Tumor treatment (*n*; %)	Surgery with tumor resection	23; 95.8	12; 100.0	11; 91.7
	Chemotherapy	15; 62.5	11; 91.7**	4; 33.3**
	Mitotane therapy	24; 100.0	12; 100.0	12; 100.0
	Radiotherapy	5; 20.8	5; 41.7*	0; 0.0*
Duration of mitotane treatment (*n*; %)	Median/mean/range (months)	18.1/13/3–46	1.1/8.2/0.3–46**	18.5/18.5/5–42**
	<1	8; 33.3	5; 41.7	3; 25.0
	1–2	13; 54.2	5; 41.7	8; 66.7
	>2	3; 12.5	2; 16.7	1, 8.3
Remission (*n*; %)	Yes	16; 66.7	8; 66.7	8, 66.7
	No	8; 14.8	4; 33.3	4; 33.3
Pre-existing illness (*n*; %)^a^	Epilepsy	0; 0.0	0; 0.0	0; 0.0
	Beckwith–Wiedemann syndrome	1; 4.2	0; 0.0	1; 8.3
	Delayed development	2; 8.3	1; 8.3	1; 8.3
	Second tumor	1; 4.2	1; 8.3	0; 0.0
	Li–Fraumeni syndrome	4; 16.7	3; 25.0	1; 8.3
	Others	3; 12.5	1; 8.3	2; 16.7

^a^ Statistical comparisons were performed between the mitotane ongoing and completed group using Fisher's exact test and Mann-Whitney U test. Asterisks and bold indicate statistically significant group differences (*P < 0.05, **P < 0.01).

Educational attainment, considered a key characteristic of the control group, was assessed in both patients (*n* = 24) and their siblings (*n* = 16). More than half of the patients (*n* = 13; 54.2%) and siblings (*n* = 9; 56.3%) had not yet obtained a school-leaving certificate, likely reflecting their young age. Nine patients (37.5%) and three siblings (18.8%) had a secondary/middle-school qualification, while one patient and three siblings had obtained a university entrance qualification.

With respect to the educational background of the patients’ families, most mothers (*n* = 14; 58.3%) and fathers (*n* = 16; 66.7%) had completed secondary or middle school. Approximately one-third of caregivers (*n* = 7; 29.2% each) held a university entrance qualification. Only three mothers and one father reported having no school-leaving certificate.

### Side effects during mitotane treatment

**Figure 1:**
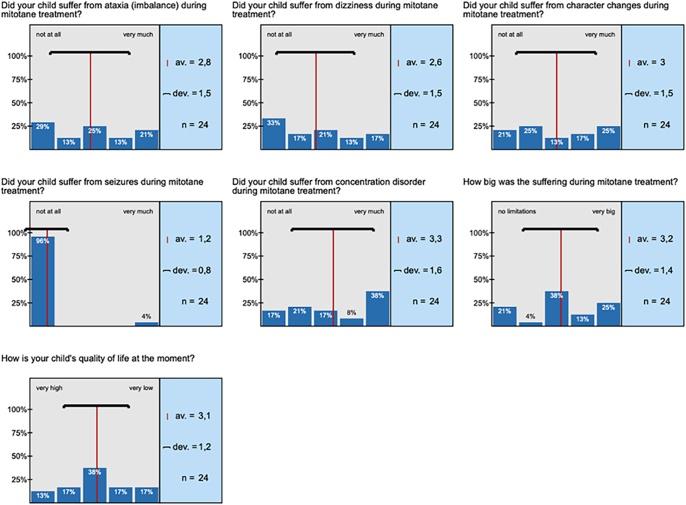
Specific side effects during mitotane therapy covering questions about the severity of ataxia, dizziness, character changes, seizures, concentration disorders, overall suffering, and quality of life at the moment; av. = mean, dev. = standard deviation, *n* = number of responses. This graphic was generated by https://evasys.de/.

Relevant adverse effects during mitotane treatment were common, with 22 patients (91.7%) reporting clinically relevant side effects and only two (8.3%) reporting none. Selected neurological side effects, assessed using a 5-point Likert scale from ‘not at all’ (1) to ‘very much’ (5), were overall rated within the moderate severity range, with slightly higher scores for concentration difficulties. Seizures were rare; for detailed distributions, see Fig. 1. All reported side effects refer to the period during mitotane therapy, irrespective of treatment status at the time of the survey.

Suffering during mitotane therapy was most often rated as moderate (35%), while current quality of life was predominantly moderate as well (38%) (see Fig. 1). Patients on active treatment reported slightly better current quality of life than those who had completed therapy, whereas the latter recalled higher treatment-related suffering.

In free-text questions addressing side effects experienced during mitotane treatment, caregivers reported neurological effects most frequently, such as balance disorder, fatigue, memory or speech disturbances, tremor, and emotional instability, with a total of 16 mentions (66.7%), followed by endocrinological (gynecomastia, precocious puberty, nocturia, and hypoglycemia; 11 mentions, 45.8%), and gastrointestinal effects (abdominal pain, loss of appetite, nausea/vomiting, and weight loss; 10 mentions, 41.7%). Here, the number of mentions refers to the total number of times any side effect within each category was reported, regardless of which specific symptom was mentioned.

Ongoing restrictions in daily life were assessed using a multiple-response question. Adrenal insufficiency was emerged as the most frequently reported limitation (*n* = 17; 70.8%), affecting nine patients currently receiving mitotane and eight patients who had already completed therapy. Among these eight patients with persistent adrenal insufficiency after treatment completion, the mean time since mitotane discontinuation was 7.1 years (0–27 years). Five had finished therapy less than 5 years ago and three more than 5 years ago. The second most common restriction was hypothyroidism, reported by 10 patients, with five patients in each treatment group. Concentration difficulties were reported by nine patients, three in the ongoing and six in the completed-therapy group. The least frequently reported restriction was developmental delay, with a total of five mentions, including two patients in the completed-therapy group.

### Auxological assessment

Length Z-scores were available for 19 patients. At diagnosis, the mean length Z-score was 0.82 ± 1.47, with most patients falling between the normal stature range (Z-score −2 to +2). A similar distribution was observed at the time of the survey, with the majority again within this range (mean length Z-score 0.15 ± 1.36).

**Figure 2:**
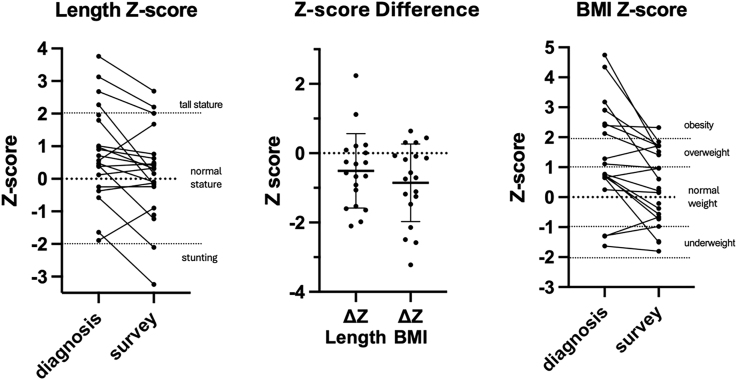
Comparison of length and BMI Z-scores at diagnosis and at the time of the survey for each patient with both values available. Nineteen patients were included for length Z-scores and twenty patients for BMI Z-scores. Z-scores were categorized as follows: length: stunting (<−2), normal stature (−2 to +2), and tall stature (>+2); BMI: mild underweight (−2 to −1), normal weight (−1 to +1), overweight (+1 to +2), and obesity (>+2).

BMI Z-scores were available for 20 patients. At diagnosis, the cohort showed a broad distribution across the BMI categories, with a mean BMI Z-score of 1.38 ± 1.74. At the time of the survey, most patients were within the normal-BMI range (Z-score −1 to +1; mean BMI Z-score 0.33 ± 1.24). Some patients fell into the overweight, obese, or mildly underweight categories at either time point. For details on Z-score development and distribution, see Fig. 2. In addition, the individual length and BMI values, including the corresponding Z-scores for each patient, are provided in Supplementary Table S1 (see the section on Supplementary materials given at the end of this article)).

### Functional participation

**Figure 3:**
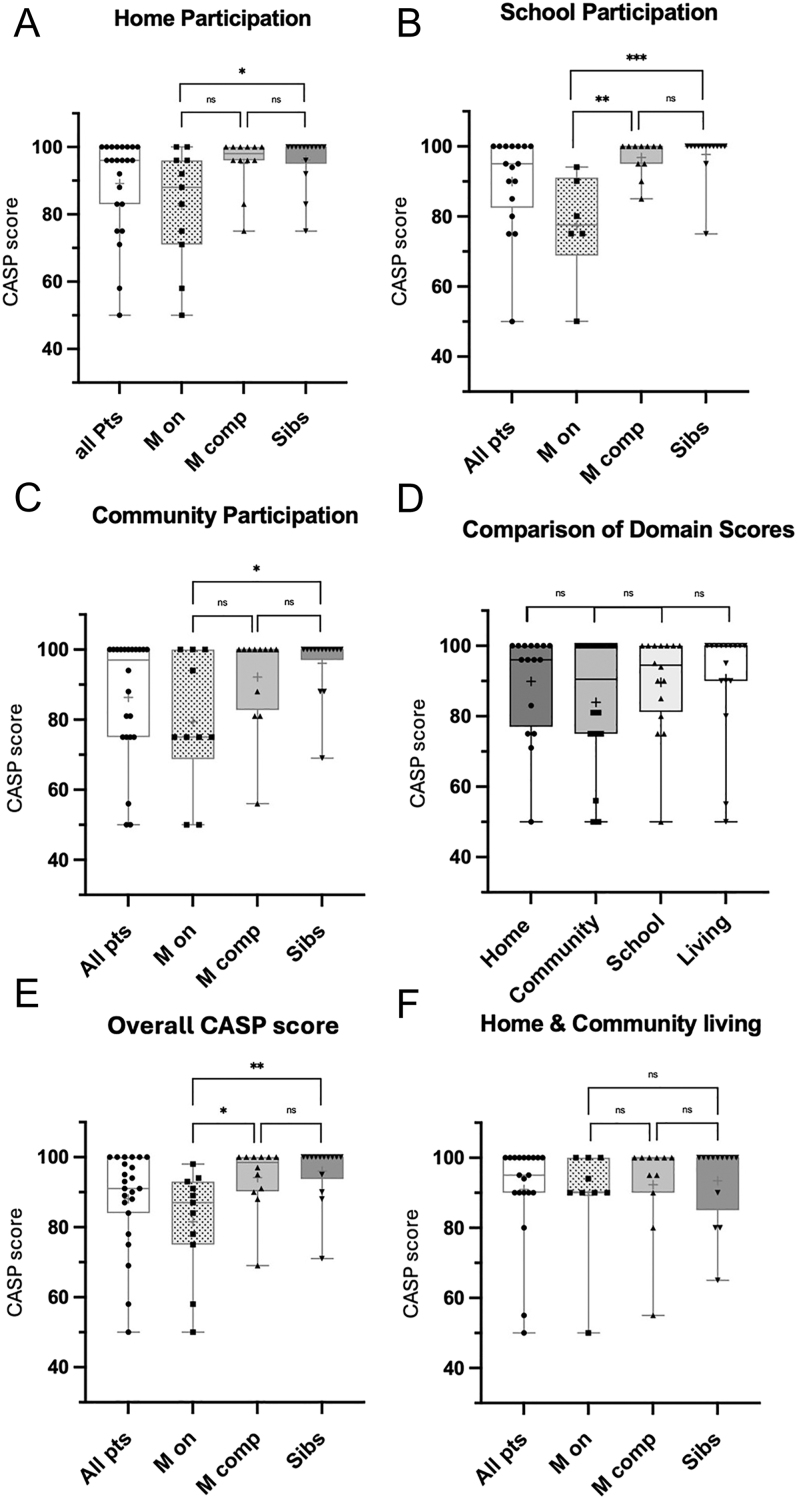
Comparison of CASP scores across all domains, divided into four groups (all patients = all Pts, mitotane ongoing = M on, mitotane completed = M comp, and siblings = Sibs). Kruskal–Wallis test, followed by Dunn’s multiple comparisons, ns = not significant. (A) Mitotane ongoing vs siblings: *P* = 0.015*; others ns (ongoing vs completed: *P* = 0.107; completed vs siblings: *P* > 0.999). (B) Mitotane ongoing vs completed: *P* = 0.006**; ongoing vs siblings: *P* = 0.001***; completed vs siblings ns. (C) Mitotane ongoing vs siblings: *P* = 0.025*; others ns (ongoing vs completed: *P* = 0.139; completed vs siblings: *P* > 0.999). (D) All group comparisons ns (ongoing vs completed: *P* > 0.999; ongoing vs siblings: *P* = 0.643; completed vs siblings: *P* > 0.999). (E) Mitotane ongoing vs completed: *P* = 0.026*; ongoing vs siblings: *P* = 0.002**, completed vs siblings: *P* > 0.999. (F) Comparison of all domains of whole cohort *n* = 24: ns; Friedman’s test: *n* = 16; x^2^(4) = 1.83; and *P* = 0.61.

Analysis of the overall CASP score showed significant differences among the three groups: patients undergoing mitotane therapy had the lowest participation levels with a mean of 81.5 ± 15.3, differing significantly from siblings (96.0 ± 8.2; *P* = 0.002**) and from patients who had completed therapy (94.2 ± 12.0; *P* = 0.025*). No significant difference was found between the completed-therapy group and siblings (see Fig. 3E). Accordingly, all subgroups fell within CASP category 2, indicating somewhat limited participation.

Domain-specific analyses confirmed this pattern in three of four domains. Children under ongoing mitotane therapy showed significantly reduced participation compared with siblings in school (77.3 ± 15.5 vs 97.7 ± 7; *P* = 0.001***), home (82.6 ± 17.1 vs 96.1 ± 7.8; *P* = 0.015*), and community participation (79.4 ± 19.1 vs 96.0 ± 9.0; *P* = 0.025*). A significant difference between the ongoing and completed therapy group was present only for school participation (77.3 ± 15.5 vs 96.8 ± 5.1; *P* = 0.006**). Home and community living showed no group differences at all. No significant differences were observed between the completed-therapy group and siblings in any domain. The Friedman test showed no significant differences across the four domains within the patient cohort (*P* = 0.61) (see Fig. 3).

For further comparison, patients who had completed mitotane therapy were stratified by time since treatment completion (<5 years, *n* = 6 vs > 5 years, *n* = 5). CASP scores differed significantly between the two groups, with lower mean scores in the <5 years group (89.2 ± 10.9) compared with the >5 years group (99.0 ± 2.2) (Mann–Whitney U test; *r* = 0.61, *P* = 0.044). In a separate analysis, the entire cohort was grouped by therapy duration (<1 year, 1–2 years, and >2 years). No difference in CASP scores were observed between these categories (Kruskal–Wallis test; *H* = 0.91, *P* = 0.635).

The exact CASP scores for the entire cohort and subgroups, categorized by participation domains, can be found in the supplementary material (see Supplementary Tables S2 and S3).

To examine whether parental education affected children’s participation, caregivers were grouped into three education levels. One parent pair had a low educational level, 14 a medium, and 8 a high educational level. Median CASP scores did not differ significantly between the three educational groups (low: 98; medium: 88.07 ± 14.44; high: 87.13 ± 13.96; Kruskal–Wallis: *H* = 0.7445, *P* = 0.689; and Dunn post hoc: all adjusted *P*-values >0.999). Thus, there is no evidence of an education-related effect on patients’ participation scores within this cohort.

**Figure 4:**
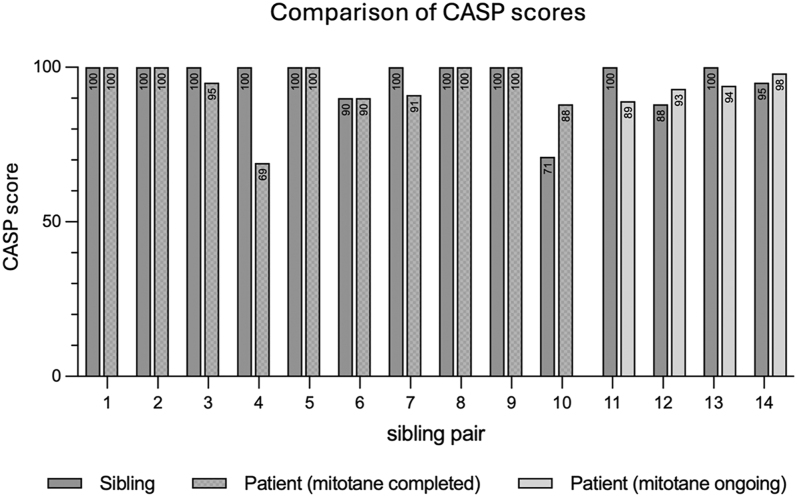
Comparison of overall CASP scores between patients (mitotane ongoing and completed) and their siblings; Wilcoxon signed-rank test: ns, *P* = 0.0328; *W* = −15.0; positive ranks: 10.5; and negative ranks: −25.5.

Individual patient–sibling comparisons showed no significant difference (Wilcoxon signed-rank test: *P* = 0.328). Although a slight negative trend was observed with patients tending to score marginally lower than siblings, the magnitude of this difference was too small to be meaningful (see Fig. 4).

In free-text responses addressing factors that interfered with patients’ participation, developmental and cognitive limitations were most frequently reported, followed by physical limitations and social–emotional challenges. Factors that supported participation most commonly included school-based support or therapeutic interventions (e.g. fine motor or speech therapy, encouragement). No child used assistive devices. Three caregivers reported practical adaptations at school or home. In contrast, siblings showed markedly fewer participation barriers, and – apart from one sibling with Joubert syndrome – no relevant limitations were described.

### Behavioral outcome

One to two items were chosen from each of the five SDQ subscales, generating nine questions in total. In general, most items showed mostly comparable results across the three groups (mitotane ongoing, mitotane completed, and siblings). Items representing *Emotional Symptoms* and *Prosocial Behavior* showed no significant overall group differences. However, three individual items across different SDQ subscales demonstrated significant group effects.

Within the *Conduct Problems* subscale, the item ‘*often loses temper*’ differed significantly: patients currently receiving mitotane tend to lose their temper more frequently than both patients who had completed therapy (*P* = 0.0343*) and siblings (*P* = 0.001**), while no difference was observed between the completed group and siblings.

In the *Hyperactivity/Inattention* subscale, the mitotane ongoing group affirmed significantly more frequently the item ‘*restless, overactive, cannot stay still for too long*’ compared with the completed group *(P* = 0.0097**). No significant differences were found between ongoing and siblings (*P* = 0.1425) or completed and siblings (*P* = 0.7292) groups.

**Figure 5:**
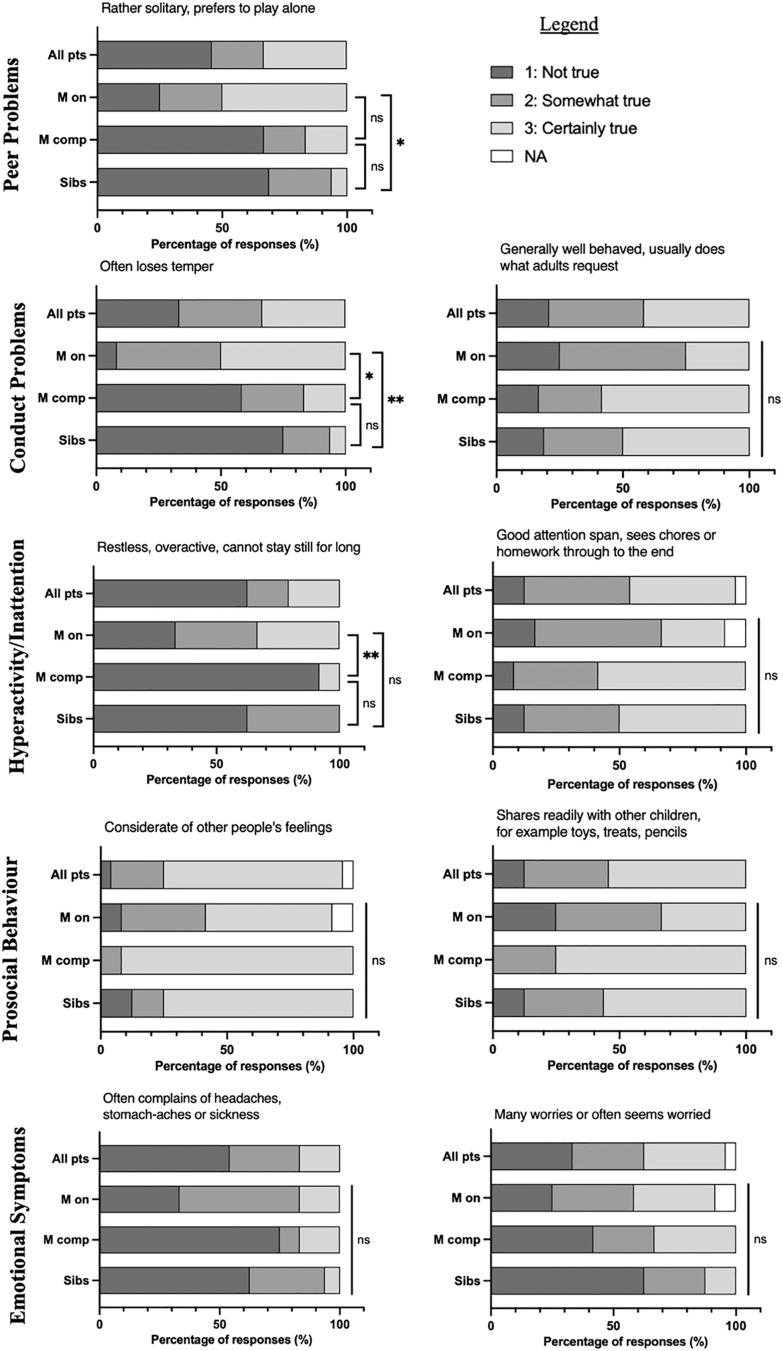
Nine items from the strength and difficulties questionnaire, showing answers in percentage (%) from all patients (all pts, *n* = 24), mitotane ongoing (M on, *n* = 12), mitotane completed (M comp, *n* = 12), and siblings (Sibs, *n* = 16). The Kruskal–Wallis test with multiple comparisons was used to compare the groups M on, M comp, Sibs, *P* < 0.05*, and *P* < 0.01**; ns = not significant.

A similar trend was found within *Peer Problems*: the item ‘*rather solitary, prefers to play alone*’ was affirmed significantly more often by the mitotane ongoing group than the siblings (*P* = 0.0231*), while the remaining pairwise comparisons were not significant (ongoing vs completed, *P* = 0.0834; completed vs siblings, *P* > 0.999) (see Fig. 5). Detailed response counts and mean scores are provided in the supplementary material (see Supplementary Table S4).

## Discussion

Mitotane is a cornerstone of treatment in advanced pediatric adrenocortical carcinoma. However, data on its long-term impact on daily functioning and psychosocial outcomes are scarce. As the first study addressing this topic, our findings suggest that while mitotane-related impairments in functional participation and psychosocial behavior are predominantly transient, endocrine sequelae – particularly adrenal insufficiency – appear to persist.

### Functional participation and behavioral outcomes

Reduced CASP scores indicate that functional participation is most impaired during ongoing mitotane therapy, whereas patients who had completed treatment demonstrated scores nearly identical to those of their siblings. This pattern suggests a normalization of functional participation over time, with only minimal residual impairments, as reflected by the slightly lower overall CASP scores in the completed-therapy group. The observed difference in CASP scores between <5 and >5 years post-therapy may suggest a time-dependent recovery of participation following mitotane treatment. In contrast, mitotane treatment duration itself does not appear to influence long-term participation in our cohort, highlighting the need for close long-term follow-up regardless of therapy duration. Furthermore, this observation suggests that factors other than treatment duration, such as disease severity or endocrine sequelae, may play an additional relevant role in shaping long-term participation. The median quality-of-life ratings in this group indicate that mitotane therapy may exert a persistent impact on perceived quality of life. Similar to functional participation, behavioral changes appear to largely normalize after treatment completion, as no differences were observed between patients who had completed therapy and their siblings.

Neurological side effects, including concentration difficulties and speech disturbances, together with SDQ-reported behavioral changes such as emotional dysregulation, restlessness, and social withdrawal – predominantly observed in patients receiving ongoing mitotane therapy – likely contributed to the observed participation limitations. Domain-specific analyses identified school participation as particularly vulnerable. This is plausible, as school settings are highly sensitive to treatment-related neurocognitive effects and behavioral alterations. Accordingly, these findings highlight the importance of early school-based support, a need further reinforced by parental free-text responses. Community participation was also affected, as a tendency toward solitary play may limit engagement in social and community activities.

These observations are consistent with existing literature showing that mitotane is frequently associated with neurological side effects, which may even lead to treatment discontinuation (7, 11, 12). Importantly, previous reviews and case reports have demonstrated that these neurological side effects are largely reversible after mitotane cessation (13, 29, 30). This finding is supported by the normalization of participation and behavioral measures observed in our cohort after treatment completion. Due to the self-reported study design, mitotane plasma levels were not available, although they represent an important factor influencing both adverse effects and functional participation. Higher mitotane plasma levels (>20 mg/L) have been associated with increased neurological toxicity (12, 31). Future prospective studies should, therefore, incorporate systematic plasma level monitoring to better evaluate its impact on long-term outcomes. In larger cohorts, it will be important to investigate whether treatment-related neurological adverse effects are associated with impaired long-term functional and behavioral outcomes and whether elevated mitotane concentrations represent a risk factor for poorer long-term function.

Patients receiving ongoing mitotane therapy more frequently underwent chemotherapy and radiotherapy, which may have further impaired participation due to cumulative treatment burden. This difference likely reflects evolving treatment protocols, as more recently treated patients – predominantly represented in the ongoing-therapy group – were exposed to more intensive multimodal approaches (7, 31). Because of the small cohort size, we did not distinguish between active disease and relapse. However, this approach may have influenced outcomes by introducing clinical heterogeneity, as disease status can affect treatment intensity, symptom burden, and psychosocial stress.

### Long-term endocrine effects

In contrast to the largely reversible functional and behavioral impairments, endocrine sequelae – most notably adrenal insufficiency – were frequently persistent. It is well established in the literature that the majority of patients develop adrenal insufficiency during mitotane therapy (11, 29, 31, 32). This pattern was confirmed in our cohort, where nine of twelve patients receiving ongoing treatment were affected. Notably, eight of twelve patients who had completed mitotane therapy appeared to have persistent adrenal insufficiency, suggesting that long-term adrenal dysfunction may be more common in pediatric patients than reported in adult populations. In adult cohorts, recovery of the hypothalamic–pituitary–adrenal axis after mitotane discontinuation has been described in the majority of cases (33, 34).

The mean interval of 7.1 years since treatment completion among patients with persistent adrenal insufficiency supports this observation and underscores the need for standardized, long-term endocrine follow-up in survivors with pACC. However, as these findings are based on parental reporting, incomplete medical knowledge or misinterpretation of survey items cannot be excluded. Therefore, these results should be confirmed in future prospective studies.

### Auxological assessment

Height and weight data were self-reported and are, therefore, subject to measurement uncertainty and potential recall bias. Accordingly, these findings are presented descriptively to provide an overview of the cohort’s auxological status. A decline in mean BMI Z-scores was observed over time. However, this may reflect the combined influence of multiple factors beyond mitotane, including endogenous steroid excess at diagnosis, effects of systemic radio- or chemotherapy, and supraphysiological glucocorticoid replacement for adrenal insufficiency (3, 4, 32, 35). Length Z-scores showed moderate variation but largely remained within the normal range at both time points. Despite these limitations, the data provide valuable preliminary insights into auxological development in children with pACC and help inform the design of future studies using standardized, prospectively collected measurements.

### Limitations

The main limitation of this study is the small sample size, which is largely attributable to the high mortality associated with advanced pACC (2, 4, 5). This significantly limited the ability to include caregivers of deceased patients. In some cases, caregivers declined participation, and in others, contacting bereaved families was considered ethically sensitive and potentially distressing.

As participation was voluntary, selection bias is likely, as caregivers of long-term survivors or those with more favorable treatment experiences may have been more inclined to respond. Given the focus on long-term outcomes in survivors, this limitation should be considered. To ensure accessibility and minimize barriers to participation in the online questionnaire (QR code-based), treating physicians provided assistance when needed, including printed versions or support with digital entry. The retrospective, observational design introduces further survivor, reporting and observer biases. Patients who had completed mitotane therapy represent long-term survivors and may, therefore, demonstrate better functional outcomes than those still undergoing treatment. The reliance on parental reporting may have led to an underestimation of severity of subjective symptoms, either unintentionally or due to social desirability. This may also explain why gastrointestinal symptoms, such as nausea, which are more directly perceived by the patient, were reported less frequently than neurological effects, which are more readily observed by caregivers.

The use of siblings as a control group is well established in similar pilot studies and represents strength of our design (36, 37). In small cohorts, siblings provide a pragmatic comparison group, as shared familial, environmental, and genetic factors improve comparability and help reduce confounding, particularly in functional and behavioral outcomes, which can be influenced by the family environment. However, this approach may introduce reporting bias, as the same caregivers provided information for both patients and controls. For larger, prospective studies, more diverse control groups, including unrelated healthy children of similar age, will be required to enable more robust comparisons.

## Conclusion and future directions

A major contribution of this study is its first structured assessment of functional participation and behavioral outcomes following mitotane therapy in pACC using validated instruments. As a multicenter pilot study, it provides important preliminary insights and lays the foundation for future, larger, prospective investigations. Our findings suggest that mitotane therapy is primarily associated with mild to moderate, treatment-related impairments in functional participation and psychosocial behavior. Importantly, these adverse effects appear to be largely transient, with functional and psychosocial measures normalizing after completion of therapy. In contrast, endocrine complications, most notably adrenal insufficiency, frequently persist beyond the treatment period. This underscores the need for standardized, long-term endocrine follow-up in affected patients. Furthermore, the persistence of endocrine sequelae highlights the importance of prospective, international studies to systematically evaluate long-term outcomes, including neuropsychological development, particularly in children treated during early childhood. Further prospective studies are planned to address remaining open questions and to expand current knowledge regarding long-term effects.

## Supplementary materials





## Declaration of interest

The authors declare that there is no conflict of interest that could be perceived as prejudicing the impartiality of the work reported.

## Funding

This work was supported by a training research grant from ‘Interdisziplinäres Zentrum für klinische Forschung (IZKF)’ awarded to MR (project number: Z-02SCP/23), a grant from the ‘Mildred Scheel Program’ awarded to VW (Project 70113303-6), and a grant from the ‘Deutsche Forschungsgemeinschaft’ (DFG) German Research Foundation (Project 314061271-TRR 205) awarded to MF. The funder had no role in study design, data collection and analysis, decision to publish, or preparation of the manuscript.

## Author contribution statement

MR and VW conceived the study; HF and VW wrote the paper and analyzed data; and HF, MR, JK, CFC, NDP, MF, SF, DJ, MM, AM, JM, SP, HSS, GD, JW, BY, MF, and VW collected data. All authors revised the manuscript.

## AI disclosure

OpenAI ChatGPT (GPT-5, GPT-4, and GPT-4o) was used during manuscript preparation exclusively for language editing and grammar improvement. All scientific content, data analysis, and interpretations were solely and independently developed and approved by the authors.
