# Supplementary material for: Functional and behavioral outcomes in pediatric adrenal carcinoma under mitotane therapy: a caregiver-reported pilot study
Source: Endocr Oncol. 2026 Jun 5;6(1):e260010. doi: 10.1530/EO-26-0010 (PMC13250649; doi:10.1530/EO-26-0010)
Supplement: Supplementary file 1 [file supplementary_materials.pdf]

Riedmeier

Mitotane_study_outcome ()
No. of responses = 24

Survey Results

Legend

Question text

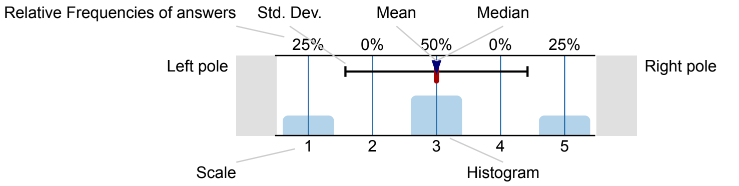

n=No. of responses
av.=Mean
md=Median
dev.=Std. Dev.
ab.=Abstention

1. General aspects of pACC treatment

1.4) What is your child's gender?

n=24

male	58,3%
female	41,7%
diverse	0%

1.5) What is the tumor stage?

n=20

I	5%
II	15%
III	30%
IV	50%

1.6) How was the tumor treated? (more than one answer is possible)

n=24

surgery with tumor resection	95,8%
chemotherapy	62,5%
mitotane therapy	100%
radiotherapy	20,8%

1.7) Is your child in remission?

n=24

yes	66,7%
no	33,3%

2. General aspects of mitotane treatment

2.2) Is your child still undergoing mitotane treatment?

n=24

yes	50%
no	50%

3. Adverse effects of mitotane treatment

3.1) Did your child develop relevant adverse effect while treatment with mitotane?

n=23

yes	91,3%
no	8,7%

3.3) Did your child suffer from ataxia (imbalance) during mitotane treatment?

not at all	1	2	3	4	5	very much	
	29,2%	12,5%	25%	12,5%	20,8%		n=24 av.=2,8 md=3 dev.=1,5

3.4) Did your child suffer from dizziness during mitotane treatment?

not at all	1	2	3	4	5	very much	
	33,3%	16,7%	20,8%	12,5%	16,7%		n=24 av.=2,6 md=2,5 dev.=1,5

3.5) Did your child suffer from character changes during mitotane treatment?

not at all	1	2	3	4	5	very much	
	20,8%	25%	12,5%	16,7%	25%		n=24 av.=3 md=3 dev.=1,5

3.6) Did your child suffer from seizures during mitotane treatment?

not at all	1	2	3	4	5	very much	
	95,8%	0%	0%	0%	4,2%		n=24 av.=1,2 md=1 dev.=0,8

3.7) Did your child suffer from concentration disorder during mitotane treatment?

not at all	1	2	3	4	5	very much	
	16,7%	20,8%	16,7%	8,3%	37,5%		n=24 av.=3,3 md=3 dev.=1,6

3.8) How big was the suffering during mitotane treatment?

no limitations	1	2	3	4	5	very big	
	20,8%	4,2%	37,5%	12,5%	25%		n=24 av.=3,2 md=3 dev.=1,4

3.9) How is your child's quality of life at the moment?

very high	1	2	3	4	5	very low	
	12,5%	16,7%	37,5%	16,7%	16,7%		n=24 av.=3,1 md=3 dev.=1,2

4. Medical history

4.1) Does your child have any pre-existing conditions, for example ...? (more than one answer is possible)

n=24

epilepsy	0%
delayed development	8,3%
second tumor	4,2%
Li Fraumeni syndrome	16,7%
others	20,8%

4.3) Does your child have ongoing restrictions in everyday lifes, for example ...? (more than one answer is possible)

n=24

concentration disorders	29,2%
delayed development	20,8%
adrenal insufficiency	70,8%
hypothyreoidism	41,7%
others	0%

5. Socio-economic backround

5.1) What are your (as mother) school-leaving qualifications?

n=24

University entrance	29,2%
Secondary/ Middle school	58,3%
No school certificate	12,5%

5.2) What are your (as father) school-leaving qualifications?

n=24

University entrance	29,2%
Secondary/ Middle school	66,7%
No school certificate	4,2%

5.3) What is your child's (patient) school-leaving qualification?

n=23

University entrance	4,3%
Secondary/ Middle school	39,1%
No school certificate	56,5%

5.4) If your child is too young to graduate, which school does he or she attend?

n=22

Kindergarden	13,6%
Elementary school	31,8%
Secondary school	27,3%
University	4,5%
None	22,7%

6. Child and Adolescent Scale of Participation (CASP): Patient

6.1) Social, play or leisure activities with family members at home (e.g., games, hobbies, "hanging out")

Age Expected – Not applicable

1	2	3	4	5
65,2%	30,4%	4,3%	0%	0%

n=23 av.=1,4 md=1 dev.=0,6

6.2) Social, play or leisure activities with friends at home (can include conversationes on the phone or internet)

Age Expected – Not applicable

1	2	3	4	5
60,9%	21,7%	13%	0%	4,3%

n=23 av.=1,7 md=1 dev.=1

6.3) Family chores, responsibilities and decisions at home (For younger children this may be getting things or putting things away when asked or helping with small parts of household chores; For older children this may be more involvement in household chores and decisions about family activities and plans)

Age Expected – Not applicable

1	2	3	4	5
65,2%	21,7%	8,7%	0%	4,3%

n=23 av.=1,6 md=1 dev.=1

6.4) Self-care activities (e.g., eating, dressing, bathing, combing or brushing hair, using the toilet)

Age Expected – Not applicable

1	2	3	4	5
69,6%	17,4%	8,7%	0%	4,3%

n=23 av.=1,5 md=1 dev.=1

6.5) Moving about in and around the home

Age Expected – Not applicable

1	2	3	4	5
69,6%	17,4%	8,7%	4,3%	0%

n=23 av.=1,5 md=1 dev.=0,8

Item	Question	Left pole	1	2	3	4	5	Right pole	Statistics
6.6)	Communicating with other children and adults at home	Age Expected	73,9%	21,7%	4,3%	0%	0%	Not applicable	n=23<br>av.=1,3<br>md=1<br>dev.=0,6
6.7)	Social, play, or leisure activities with friends in the neighborhood and community (e.g., casual games, “hanging out,” going to public places like a movie theater, park or restaurant)	Age Expected	52,2%	21,7%	17,4%	0%	8,7%	Not applicable	n=23<br>av.=1,9<br>md=1<br>dev.=1,2
6.8)	Structured events and activities in the neighborhood and community (e.g., team sports, clubs, holiday or religious events, concerts, parades and fairs)	Age Expected	50%	22,7%	18,2%	0%	9,1%	Not applicable	n=22<br>av.=2<br>md=1,5<br>dev.=1,3
6.9)	Moving around the neighborhood and community (e.g., public buildings, parks, restaurants, movies) [Please consider your child’s primary way of moving around, NOT his or her use of transportation]	Age Expected	52,2%	26,1%	13%	0%	8,7%	Not applicable	n=23<br>av.=1,9<br>md=1<br>dev.=1,2
6.10)	Communicating with other children and adults in the neighborhood and community	Age Expected	65,2%	21,7%	8,7%	0%	4,3%	Not applicable	n=23<br>av.=1,6<br>md=1<br>dev.=1
6.11)	Educational (academic) activities with other children in his or her classroom at school	Age expected	50%	25%	10%	0%	15%	Not applicable	n=20<br>av.=2,1<br>md=1,5<br>dev.=1,4
6.12)	Social, play and recreational activities with other children at school (e.g., “hanging out,” sports, clubs, hobbies, creative arts, lunchtime or recess activities)	Age expected	45%	25%	15%	0%	15%	Not applicable	n=20<br>av.=2,2<br>md=2<br>dev.=1,4
6.13)	Moving around at school (e.g., to get to and use bathroom, playground, cafeteria, library or other rooms and things that are available to other children his or her age)	Age expected	60%	20%	5%	0%	15%	Not applicable	n=20<br>av.=1,9<br>md=1<br>dev.=1,4
6.14)	Using educational materials and equipment that are available to other children in his or her classroom/s or that have been modified for your child (e.g., books, computers, chairs and desks)	Age expected	65%	10%	5%	0%	20%	Not applicable	n=20<br>av.=2<br>md=1<br>dev.=1,6
6.15)	Communicating with other children and adults at school	Age expected	70%	10%	5%	0%	15%	Not applicable	n=20<br>av.=1,8<br>md=1<br>dev.=1,5
6.16)	Household activities (e.g., preparing some meals, doing laundry, washing dishes)	Age expected	69,6%	13%	4,3%	0%	13%	Not applicable	n=23<br>av.=1,7<br>md=1<br>dev.=1,4
6.17)	Shopping and managing money (e.g., shopping at stores, figuring out correct change)	Age expected	65,2%	8,7%	8,7%	0%	17,4%	Not applicable	n=23<br>av.=2<br>md=1<br>dev.=1,6
6.18)	Managing daily schedule (e.g., doing and completing daily activities on time; organizing and adjusting time and schedule when needed)	Age expected	47,8%	26,1%	8,7%	0%	17,4%	Not applicable	n=23<br>av.=2,1<br>md=2<br>dev.=1,5

6.19) Using transportation to get around in the community (e.g., to and from school, work, social or leisure activities) [Driving vehicle or using public transportation]

Age expected 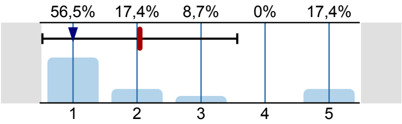 Not applicable

n=23
av.=2
md=1
dev.=1,5

6.20) Work activities and responsibilities (e.g., completion of work tasks, punctuality, attendance and getting along with supervisors and co-workers)

Age expected 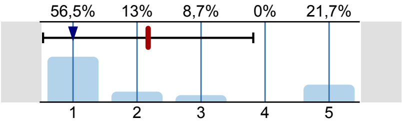 Not applicable

n=23
av.=2,2
md=1
dev.=1,6

6.23) Does your child currently use any assistive devices or equipment to help him or her participate (e.g., adapted eating utensils, shower chair, note-taker for school, daily planner, computer)?

yes 0%
no 100%

n=23

6.25) Have any changes been made to your home, community or the school (or work) setting to help your child participate (e.g., rearranging furniture and materials, adjusting lighting or noise levels, building a ramp or other physical structures)?

yes 13%
no 87%

n=23

7. SD-Questionnaire: Patient

7.1) Considerate of other people's feelings

Not True 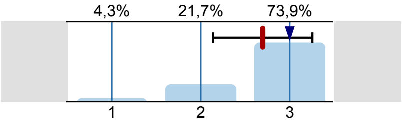 Certainly True

n=23
av.=2,7
md=3
dev.=0,6

7.2) Restless, overactive, cannot stay still for long

Not True 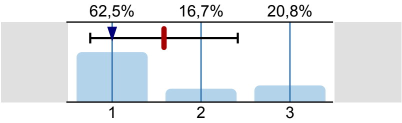 Certainly True

n=24
av.=1,6
md=1
dev.=0,8

7.3) Often complains of headaches, stomach-aches or sickness

Not True 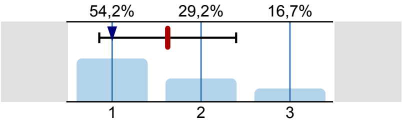 Certainly True

n=24
av.=1,6
md=1
dev.=0,8

7.4) Shares readily with other children, for example toys, treats, pencils

Not True 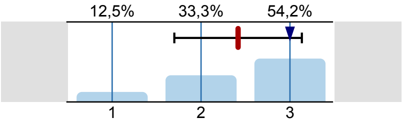 Certainly True

n=24
av.=2,4
md=3
dev.=0,7

7.5) Often loses temper

Not True 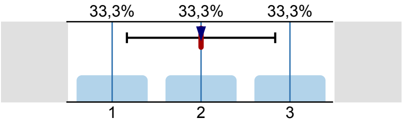 Certainly True

n=24
av.=2
md=2
dev.=0,8

7.6) Rather solitary, prefers to play alone

Not True 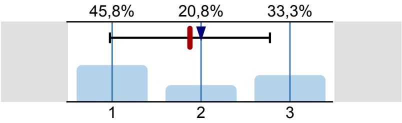 Certainly True

n=24
av.=1,9
md=2
dev.=0,9

7.7) Generally well behaved, usually does what adults request

Not True 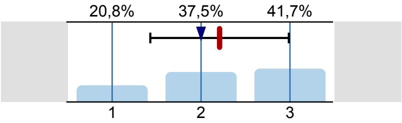 Certainly True

n=24
av.=2,2
md=2
dev.=0,8

7.8) Many worries or often seems worried

Not True 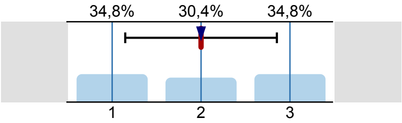 Certainly True

n=23
av.=2
md=2
dev.=0,9

7.9) Good attention span, sees chores or homework through the end

Not True	1	2	3	Certainly True	
	13%	43,5%	43,5%		n=23<br>av.=2,3<br>md=2<br>dev.=0,7

8. Questionnaire for the patient's sibling

8.2) What is the sibling's school-leaving qualification?

University entrance	20%	n=15
Secondary/ Middle school	20%	
No school certificate	60%	

8.3) If the sibling is too young to graduate, which school does he or she attend?

Kindergarden	30%	n=10
Elementary school	20%	
Secondary school	40%	
None	10%	
High School	0%	

9. CASP Sibling

Item	Age Expected 1	2	3	4	5 Not applicable	Statistics
9.1) Social, play or leisure activities with family members at home (e.g., games, hobbies, "hanging out")	80%	13,3%	0%	0%	6,7%	n=15<br>av.=1,4<br>md=1<br>dev.=1,1
9.2) Social, play or leisure activities with friends at home (can include conversationes on the phone or internet)	86,7%	6,7%	0%	0%	6,7%	n=15<br>av.=1,3<br>md=1<br>dev.=1
9.3) Family chores, responsibilities and decisions at home (For younger children this may be getting things or putting things away when asked or helping with small parts of household chores; For older children this may be more involvement in household chores and decisions about family activities and plans)	73,3%	13,3%	6,7%	0%	6,7%	n=15<br>av.=1,5<br>md=1<br>dev.=1,1
9.4) Self-care activities (e.g., eating, dressing, bathing, combing or brushing hair, using the toilet)	80%	13,3%	0%	0%	6,7%	n=15<br>av.=1,4<br>md=1<br>dev.=1,1
9.5) Moving in and around the home	80%	13,3%	0%	0%	6,7%	n=15<br>av.=1,4<br>md=1<br>dev.=1,1
9.6) Communicating with other children and adults at home	80%	13,3%	0%	0%	6,7%	n=15<br>av.=1,4<br>md=1<br>dev.=1,1
9.7) Social, play, or leisure activities with friends in the neighborhood and community (e.g., casual games, “hanging out,” going to public places like a movie theater, park or restaurant)	86,7%	0%	6,7%	0%	6,7%	n=15<br>av.=1,4<br>md=1<br>dev.=1,1

No.	Item	Left pole	1	2	3	4	5	Right pole	Statistics
9.8)	Structured events and activities in the neighborhood and community (e.g., team sports, clubs, holiday or religious events, concerts, parades and fairs)	Age Expected	66,7%	20%	0%	0%	13,3%	Not applicable	n=15 av.=1,7 md=1 dev.=1,4
9.9)	Moving around the neighborhood and community (e. g., public buildings, parks, restaurants, movies) [Please consider your child's primary way of moving around, NOT his or her use of transportation]	Age Expected	66,7%	20%	0%	0%	13,3%	Not applicable	n=15 av.=1,7 md=1 dev.=1,4
9.10)	Communicating with other children and adults in the neighborhood and community	Age Expected	80%	6,7%	0%	0%	13,3%	Not applicable	n=15 av.=1,6 md=1 dev.=1,4
9.11)	Educational (academic) activities with other children in his or her classroom at school	Age expected	80%	6,7%	0%	0%	13,3%	Not applicable	n=15 av.=1,6 md=1 dev.=1,4
9.12)	Social, play and recreational activities with other children at school (e.g., "hanging out," sports, clubs, hobbies, creative arts, lunchtime or recess activities)	Age expected	73,3%	13,3%	0%	0%	13,3%	Not applicable	n=15 av.=1,7 md=1 dev.=1,4
9.13)	Moving around at school (e.g., to get to and use bathroom, playground, cafeteria, library or other rooms and things that are available to other children his or her age)	Age expected	80%	6,7%	0%	0%	13,3%	Not applicable	n=15 av.=1,6 md=1 dev.=1,4
9.14)	Using educational materials and equipment that are available to other children in his or her classroom/s or that have been modified for your child (e.g., books, computers, chairs and desks)	Age expected	80%	6,7%	0%	0%	13,3%	Not applicable	n=15 av.=1,6 md=1 dev.=1,4
9.15)	Communicating with other children and adults at school	Age expected	80%	6,7%	0%	0%	13,3%	Not applicable	n=15 av.=1,6 md=1 dev.=1,4
9.16)	Household activities (e.g., preparing some meals, doing laundry, washing dishes)	Age expected	66,7%	6,7%	13,3%	0%	13,3%	Not applicable	n=15 av.=1,9 md=1 dev.=1,5
9.17)	Shopping and managing money (e.g., shopping at stores, figuring out correct change)	Age expected	66,7%	20%	0%	0%	13,3%	Not applicable	n=15 av.=1,7 md=1 dev.=1,4
9.18)	Managing daily schedule (e.g., doing and completing daily activities on time; organizing and adjusting time and schedule when needed)	Age expected	66,7%	20%	0%	0%	13,3%	Not applicable	n=15 av.=1,7 md=1 dev.=1,4
9.19)	Using transportation to get around in the community (e.g., to and from school, work, social or leisure activities) [Driving vehicle or using public transportation]	Age expected	73,3%	6,7%	6,7%	0%	13,3%	Not applicable	n=15 av.=1,7 md=1 dev.=1,4
9.20)	Work activities and responsibilities (e.g., completion of work tasks, punctuality, attendance and getting along with supervisors and co-workers)	Age expected	66,7%	20%	0%	0%	13,3%	Not applicable	n=15 av.=1,7 md=1 dev.=1,4

9.23) Does your child currently use any assistive devices or equipment to help him or her participate (e.g., adapted eating utensils, shower chair, note-taker for school, daily planner, computer)?

yes	0%	n=15
no	100%	

9.25) Have any changes been made to your home, community or the school (or work) setting to help your child participate (e.g., rearranging furniture and materials, adjusting lighting or noise levels, building a ramp or other physical structures)?

yes	6,7%	n=15
no	93,3%	

10. SD-Questionnaire Sibling

10.1) Considerate of other people's feelings

Not True
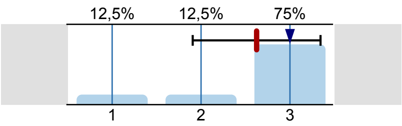

Certainly True

n=16
av.=2,6
md=3
dev.=0,7

10.2) Restless, overactive, cannot stay still for long

Not True
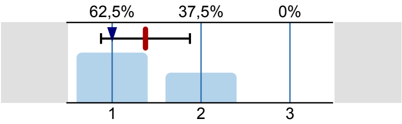

Certainly True

n=16
av.=1,4
md=1
dev.=0,5

10.3) Often complains of headaches, stomach-aches or sickness

Not True
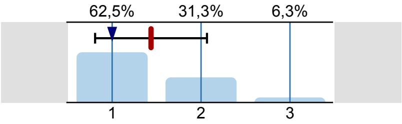

Certainly True

n=16
av.=1,4
md=1
dev.=0,6

10.4) Shares readily with other children, for example toys, treats, pencils

Not True
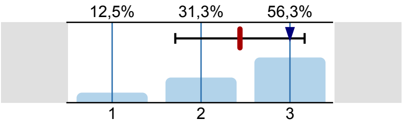

Certainly True

n=16
av.=2,4
md=3
dev.=0,7

10.5) Often loses temper

Not True
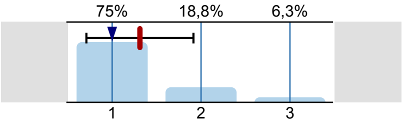

Certainly True

n=16
av.=1,3
md=1
dev.=0,6

10.6) Rather solitary, prefers to play alone

Not True
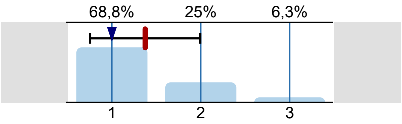

Certainly True

n=16
av.=1,4
md=1
dev.=0,6

10.7) Generally well behaved, usually does what adults request

Not True
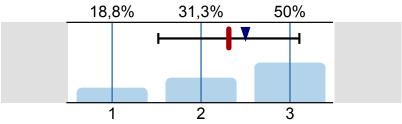

Certainly True

n=16
av.=2,3
md=2,5
dev.=0,8

10.8) Many worries or often seems worried

Not True
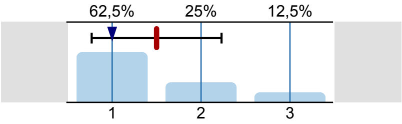

Certainly True

n=16
av.=1,5
md=1
dev.=0,7

10.9) Good attention span, sees chores or homework through to the end

Not True
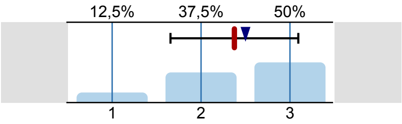

Certainly True

n=16
av.=2,4
md=2,5
dev.=0,7

Histogram for scaled questions

Did your child suffer from ataxia (imbalance) during mitotane treatment?

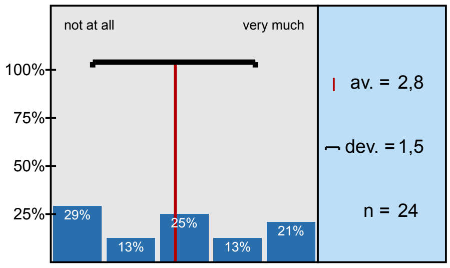

Did your child suffer from dizziness during mitotane treatment?

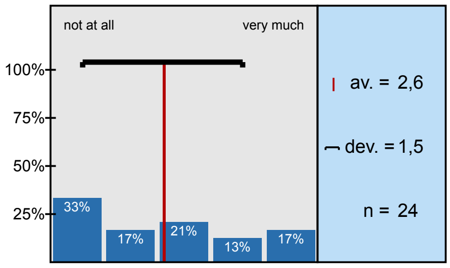

Did your child suffer from character changes during mitotane treatment?

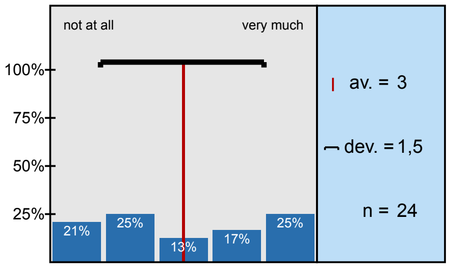

Did your child suffer from seizures during mitotane treatment?

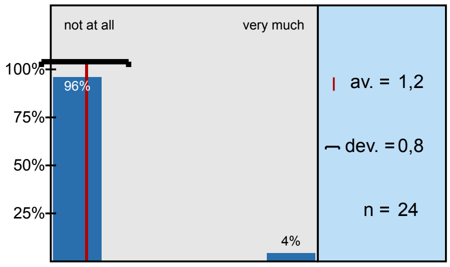

Did your child suffer from concentration disorder during mitotane treatment?

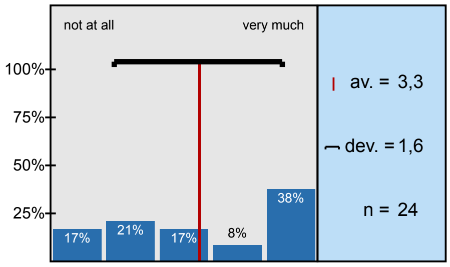

How big was the suffering during mitotane treatment?

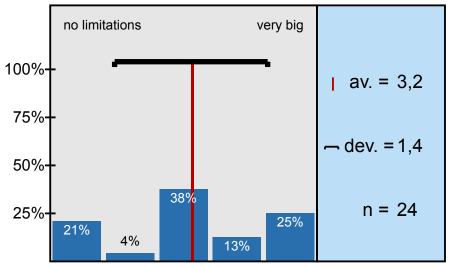

How is your child's quality of life at the moment?

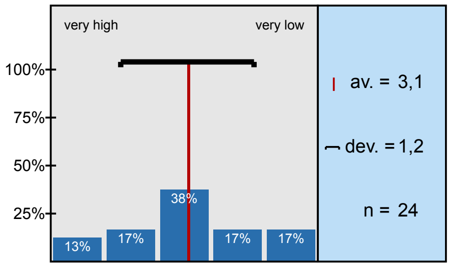

Social, play or leisure activities with family members at home (e.g., games, hobbies, "hanging out")

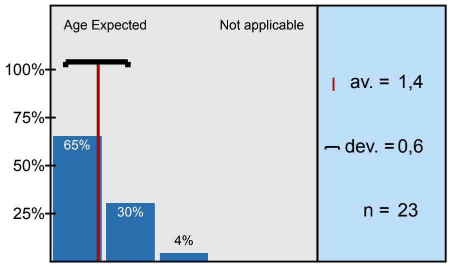

Social, play or leisure activities with friends at home (can include conversationes on the phone or internet)

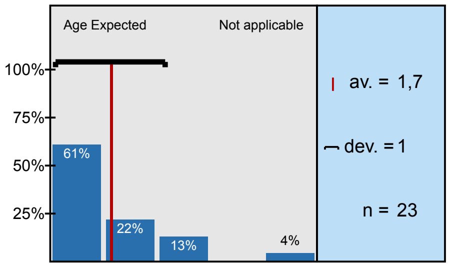

Family chores, responsibilities and decisions at home (For younger children this may be getting things or

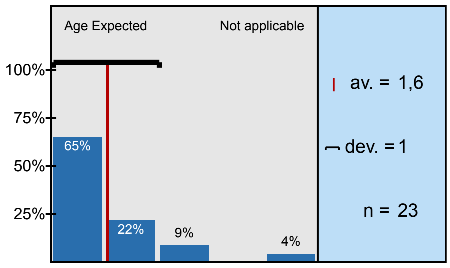

Self-care activities (e.g., eating, dressing, bathing, combing or brushing hair, using the toilet)

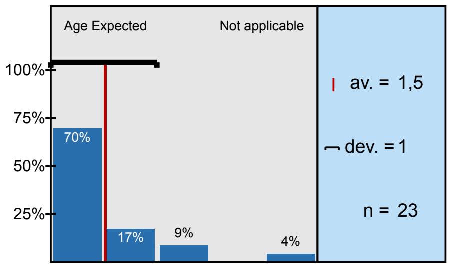

Moving about in and around the home

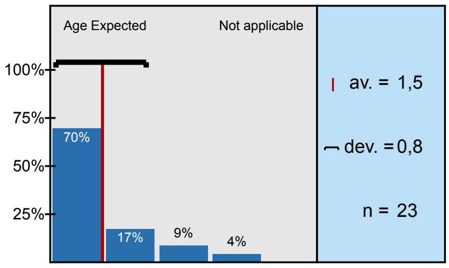

Communicating with other children and adults at home

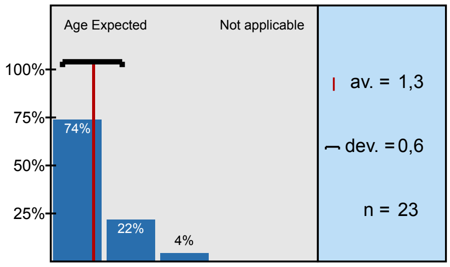

Social, play, or leisure activities with friends in the neighborhood and community (e.g., casual games,

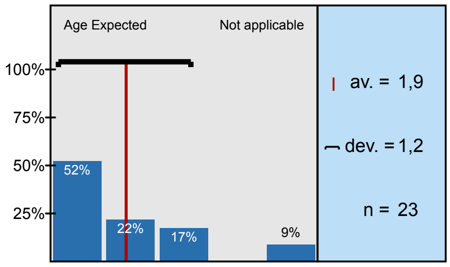

Structured events and activities in the neighborhood and community (e.g., team sports, clubs, holiday or

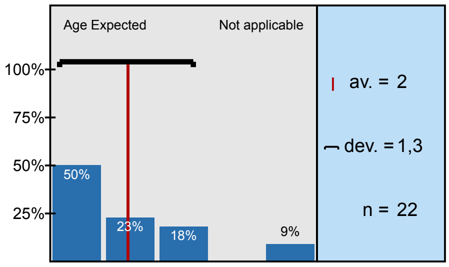

Moving around the neighborhood and community (e. g., public buildings, parks, restaurants, movies)

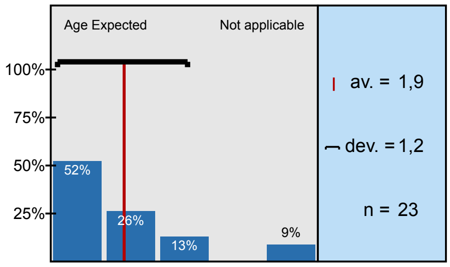

Communicating with other children and adults in the neighborhood and community

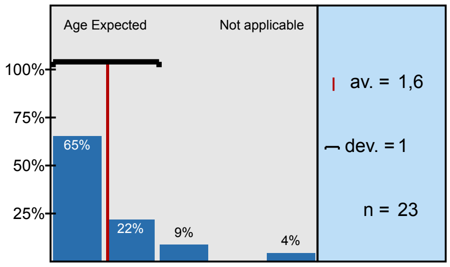

Educational (academic) activities with other children in his or her classroom at school

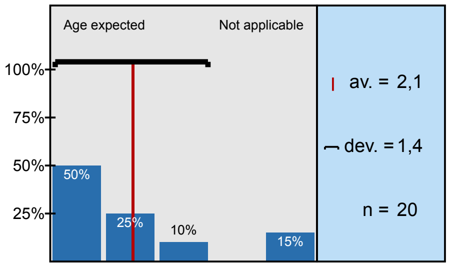

Social, play and recreational activities with other children at school (e.g., “hanging out,” sports, clubs,

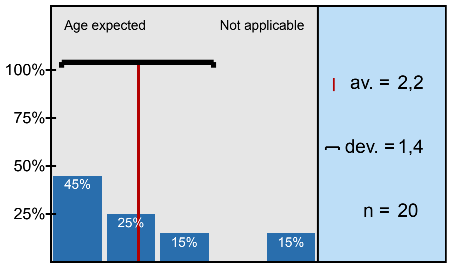

Moving around at school (e.g., to get to and use bathroom, playground, cafeteria, library or other

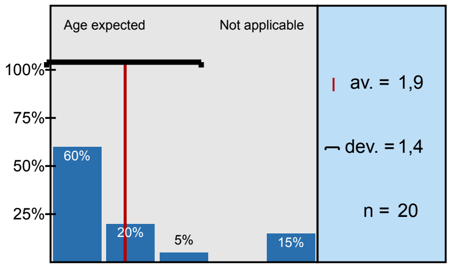

Using educational materials and equipment that are available to other children in his or her classroom/s or

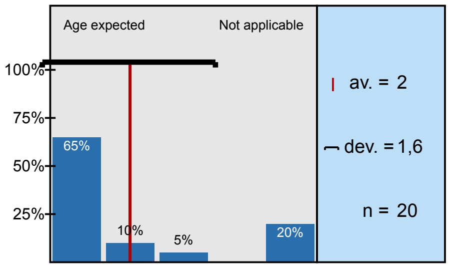

Communicating with other children and adults at school

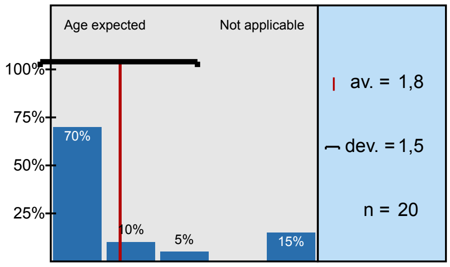

Household activities (e.g., preparing some meals, doing laundry, washing dishes)

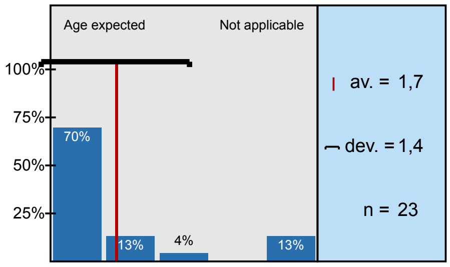

Shopping and managing money (e.g., shopping at stores, figuring out correct change)

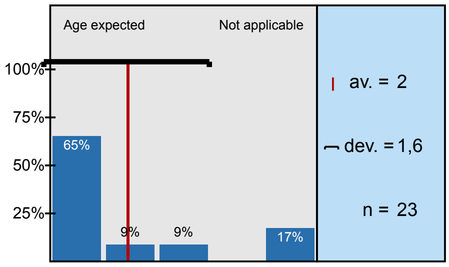

Managing daily schedule (e.g., doing and completing daily activities on time; organizing and adjusting time

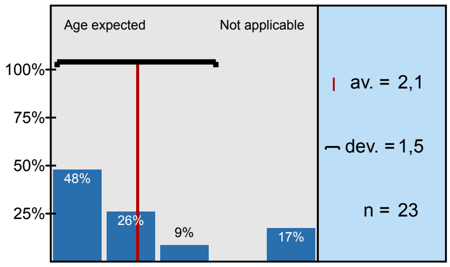

Using transportation to get around in the community (e.g., to and from school, work, social or leisure

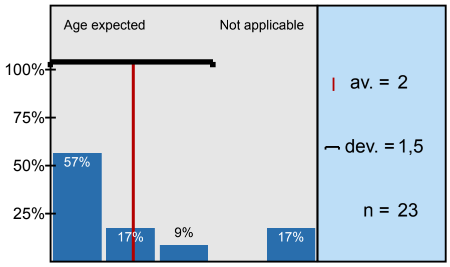

Work activities and responsibilities (e.g., completion of work tasks, punctuality, attendance and getting

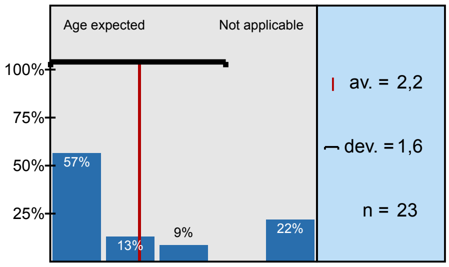

Considerate of other people's feelings

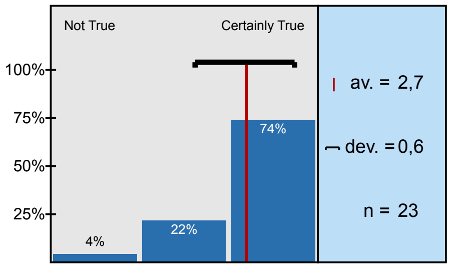

Restless, overactive, cannot stay still for long

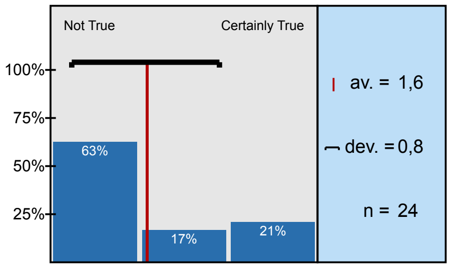

Often complains of headaches, stomach-aches or sickness

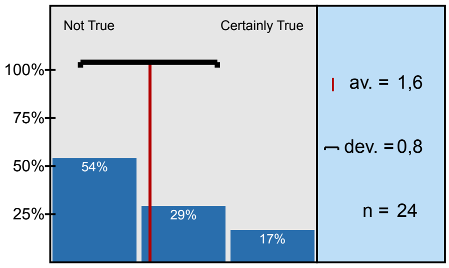

Shares readily with other children, for example toys, treats, pencils

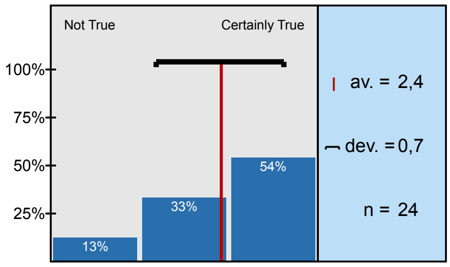

Often loses temper

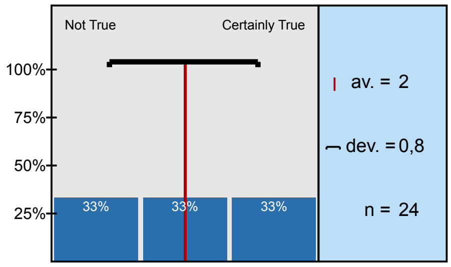

Rather solitary, prefers to play alone

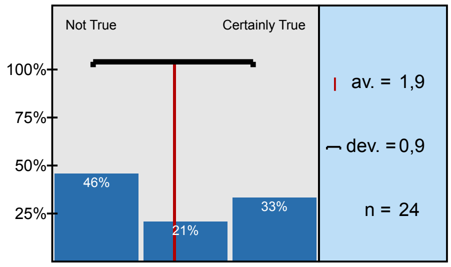

Generally well behaved, usually does what adults request

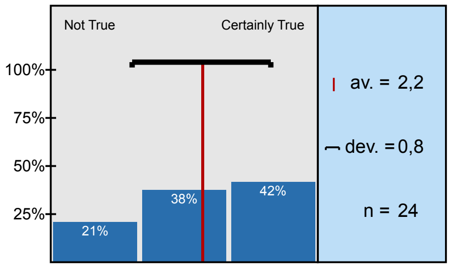

Many worries or often seems worried

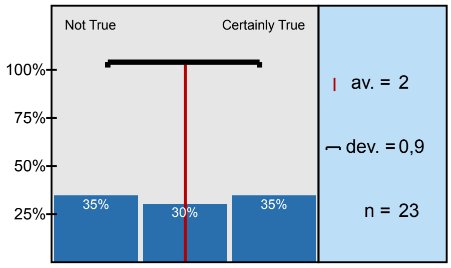

Good attention span, sees chores or homework through to the end

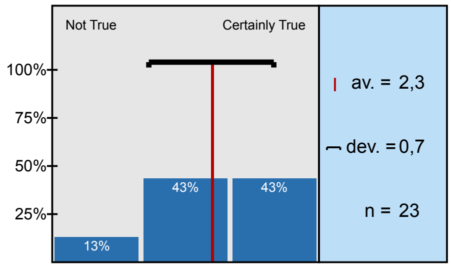

Social, play or leisure activities with family members at home (e.g., games, hobbies, "hanging out")

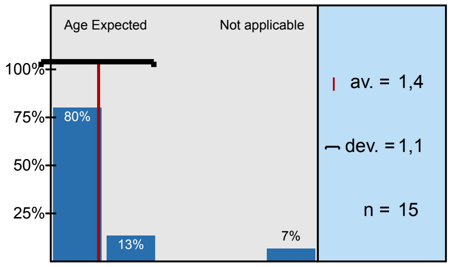

Social, play or leisure activities with friends at home (can include conversationes on the phone or internet)

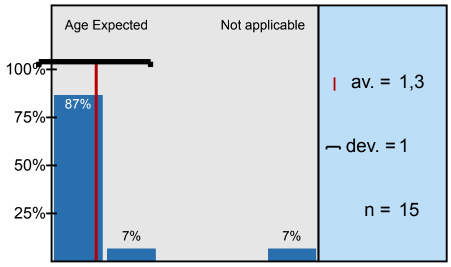

Family chores, responsibilities and decisions at home (For younger children this may be getting things or

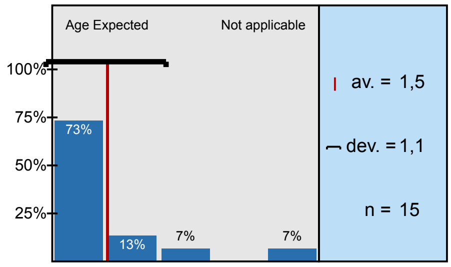

Self-care activities (e.g., eating, dressing, bathing, combing or brushing hair, using the toilet)

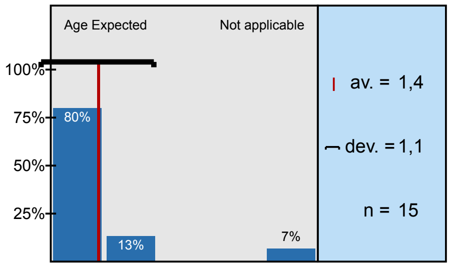

Moving in and around the home

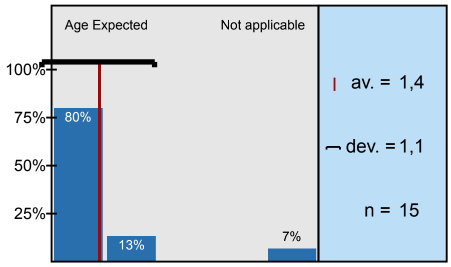

Communicating with other children and adults at home

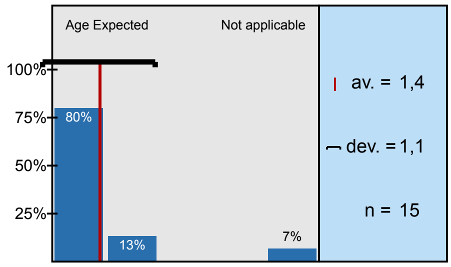

Social, play, or leisure activities with friends in the neighborhood and community (e.g., casual games,

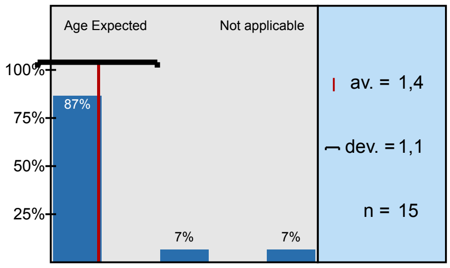

Structured events and activities in the neighborhood and community (e.g., team sports, clubs, holiday or

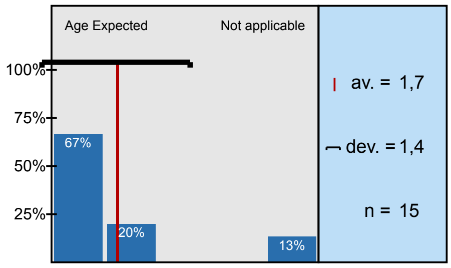

Moving around the neighborhood and community (e. g., public buildings, parks, restaurants, movies)

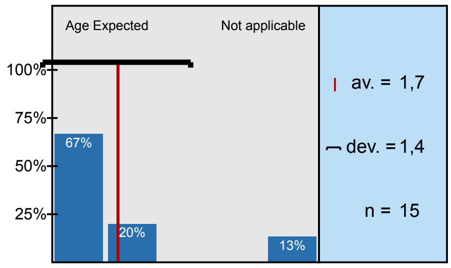

Communicating with other children and adults in the neighborhood and community

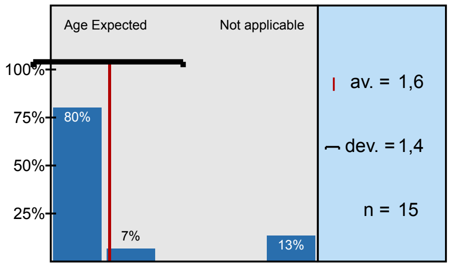

Educational (academic) activities with other children in his or her classroom at school

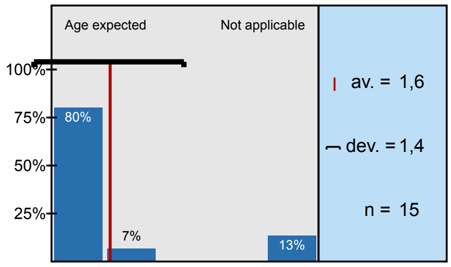

Social, play and recreational activities with other children at school (e.g., "hanging out," sports, clubs,

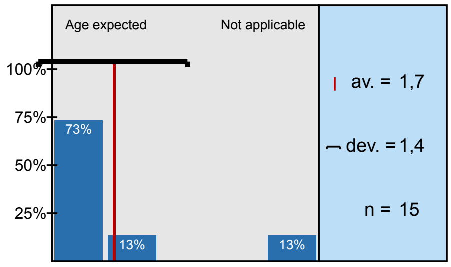

Moving around at school (e.g., to get to and use bathroom, playground, cafeteria, library or other

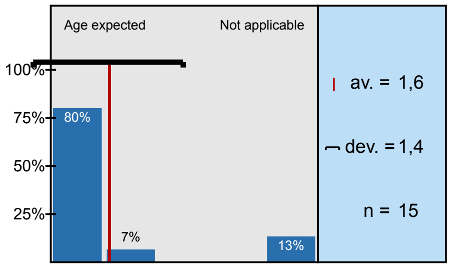

Using educational materials and equipment that are available to other children in his or her classroom/s or

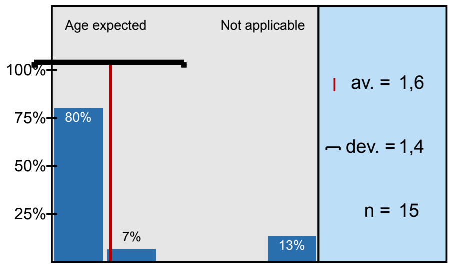

Communicating with other children and adults at school

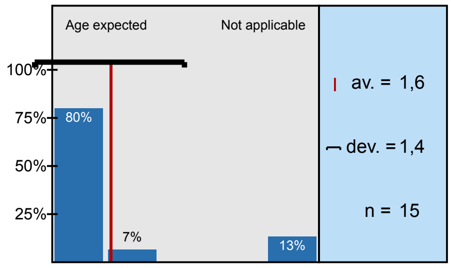

Household activities (e.g., preparing some meals, doing laundry, washing dishes)

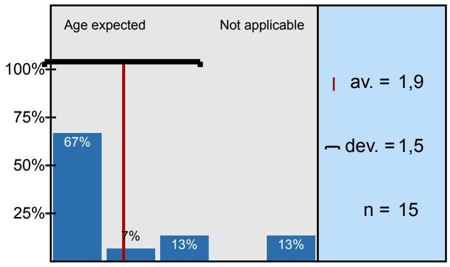

Shopping and managing money (e.g., shopping at stores, figuring out correct change)

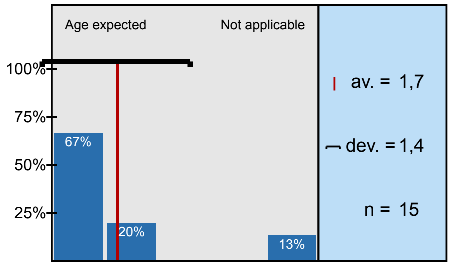

Managing daily schedule (e.g., doing and completing daily activities on time; organizing and adjusting time

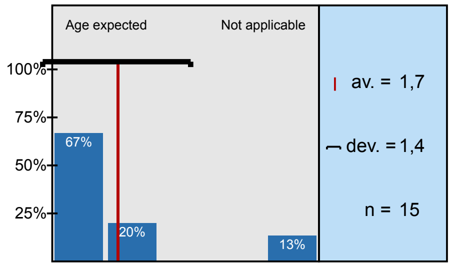

Using transportation to get around in the community (e.g., to and from school, work, social or leisure

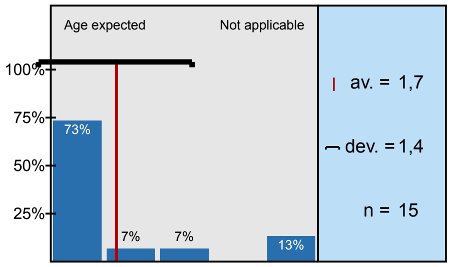

Work activities and responsibilities (e.g., completion of work tasks, punctuality, attendance and getting

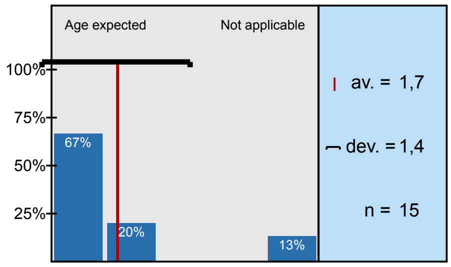

Considerate of other people's feelings

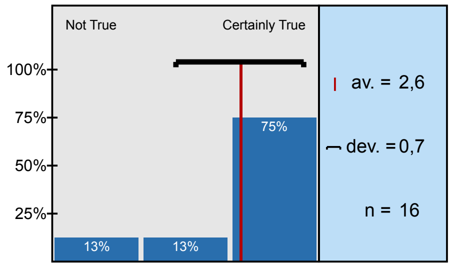

Restless, overactive, cannot stay still for long

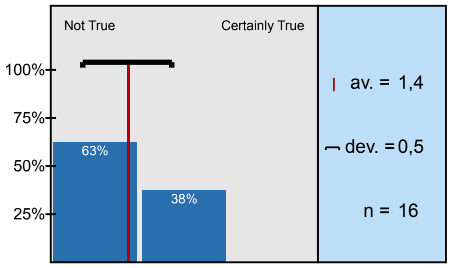

Often complains of headaches, stomach-aches or sickness

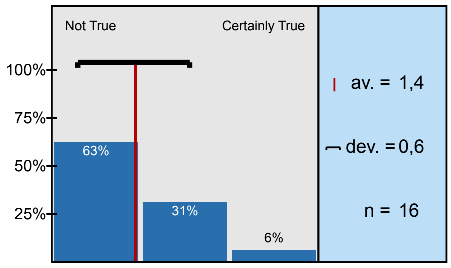

Shares readily with other children, for example toys, treats, pencils

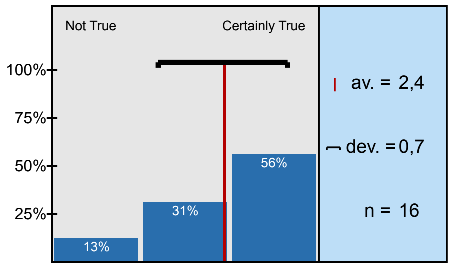

Often loses temper

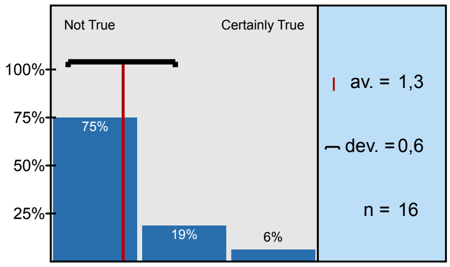

Rather solitary, prefers to play alone

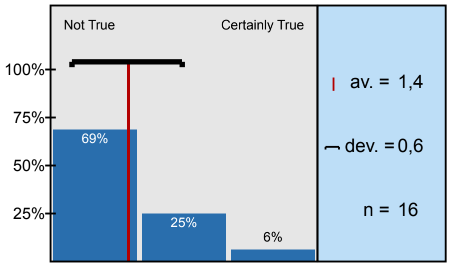

Generally well behaved, usually does what adults request

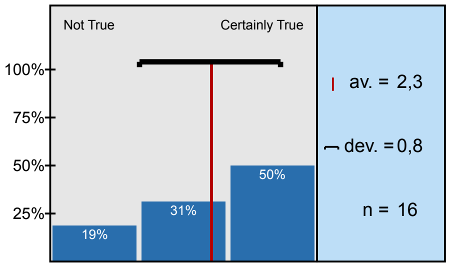

Many worries or often seems worried

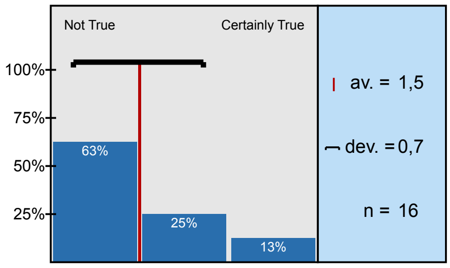

Good attention span, sees chores or homework through to the end

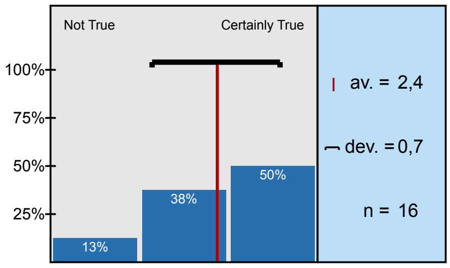

Profile

Subunit:	Kinderklinik
Name of the project:	Kinderklinik (Riedmeier)
Name of the topic: (Name of the survey)	Mitotane_study_outcome

Values used in the profile line: Mean

3. Adverse effects of mitotane treatment

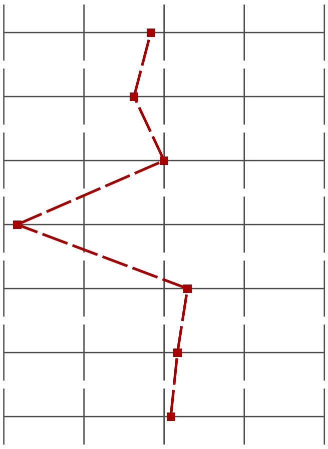

	Question	Left pole	Right pole	n	av.	md	dev.
3.3)	Did your child suffer from ataxia (imbalance) during mitotane treatment?	not at all	very much	n=24	av.=2,8	md=3	dev.=1,5
3.4)	Did your child suffer from dizziness during mitotane treatment?	not at all	very much	n=24	av.=2,6	md=2,5	dev.=1,5
3.5)	Did your child suffer from character changes during mitotane treatment?	not at all	very much	n=24	av.=3	md=3	dev.=1,5
3.6)	Did your child suffer from seizures during mitotane treatment?	not at all	very much	n=24	av.=1,2	md=1	dev.=0,8
3.7)	Did your child suffer from concentration disorder during mitotane treatment?	not at all	very much	n=24	av.=3,3	md=3	dev.=1,6
3.8)	How big was the suffering during mitotane treatment?	no limitations	very big	n=24	av.=3,2	md=3	dev.=1,4
3.9)	How is your child's quality of life at the moment?	very high	very low	n=24	av.=3,1	md=3	dev.=1,2

6. Child and Adolescent Scale of Participation (CASP): Patient

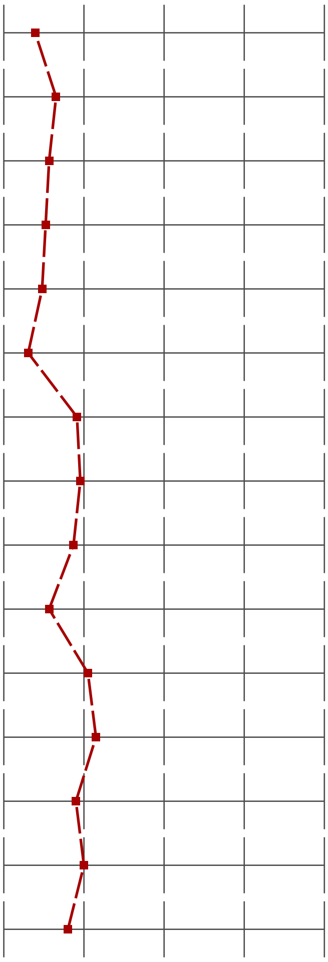

	Question	Left pole	Right pole	n	av.	md	dev.
6.1)	Social, play or leisure activities with family members at home (e.g., games, hobbies, "hanging out")	Age Expected	Not applicable	n=23	av.=1,4	md=1	dev.=0,6
6.2)	Social, play or leisure activities with friends at home (can include conversationes on the phone or internet)	Age Expected	Not applicable	n=23	av.=1,7	md=1	dev.=1
6.3)	Family chores, responsibilities and decisions at home (For younger children this may be getting things or putting things away when asked or	Age Expected	Not applicable	n=23	av.=1,6	md=1	dev.=1
6.4)	Self-care activities (e.g., eating, dressing, bathing, combing or brushing hair, using the toilet)	Age Expected	Not applicable	n=23	av.=1,5	md=1	dev.=1
6.5)	Moving about in and around the home	Age Expected	Not applicable	n=23	av.=1,5	md=1	dev.=0,8
6.6)	Communicating with other children and adults at home	Age Expected	Not applicable	n=23	av.=1,3	md=1	dev.=0,6
6.7)	Social, play, or leisure activities with friends in the neighborhood and community (e.g., casual games, “hanging out,” going to public places	Age Expected	Not applicable	n=23	av.=1,9	md=1	dev.=1,2
6.8)	Structured events and activities in the neighborhood and community (e.g., team sports, clubs, holiday or religious events,	Age Expected	Not applicable	n=22	av.=2	md=1,5	dev.=1,3
6.9)	Moving around the neighborhood and community (e.g., public buildings, parks, restaurants, movies) [Please consider your	Age Expected	Not applicable	n=23	av.=1,9	md=1	dev.=1,2
6.10)	Communicating with other children and adults in the neighborhood and community	Age Expected	Not applicable	n=23	av.=1,6	md=1	dev.=1
6.11)	Educational (academic) activities with other children in his or her classroom at school	Age expected	Not applicable	n=20	av.=2,1	md=1,5	dev.=1,4
6.12)	Social, play and recreational activities with other children at school (e.g., “hanging out,” sports, clubs, hobbies, creative arts, lunchtime	Age expected	Not applicable	n=20	av.=2,2	md=2	dev.=1,4
6.13)	Moving around at school (e.g., to get to and use bathroom, playground, cafeteria, library or other rooms and things that are available to	Age expected	Not applicable	n=20	av.=1,9	md=1	dev.=1,4
6.14)	Using educational materials and equipment that are available to other children in his or her classroom/s or that have been modified for	Age expected	Not applicable	n=20	av.=2	md=1	dev.=1,6
6.15)	Communicating with other children and adults at school	Age expected	Not applicable	n=20	av.=1,8	md=1	dev.=1,5

6.16)	Household activities (e.g., preparing some meals, doing laundry, washing dishes)	Age expected	Not applicable	n=23	av.=1,7	md=1	dev.=1,4
6.17)	Shopping and managing money (e.g., shopping at stores, figuring out correct change)	Age expected	Not applicable	n=23	av.=2	md=1	dev.=1,6
6.18)	Managing daily schedule (e.g., doing and completing daily activities on time; organizing and adjusting time and schedule when needed)	Age expected	Not applicable	n=23	av.=2,1	md=2	dev.=1,5
6.19)	Using transportation to get around in the community (e.g., to and from school, work, social or leisure activities) [Driving vehicle or	Age expected	Not applicable	n=23	av.=2	md=1	dev.=1,5
6.20)	Work activities and responsibilities (e.g., completion of work tasks, punctuality, attendance and getting along with supervisors	Age expected	Not applicable	n=23	av.=2,2	md=1	dev.=1,6

7. SD-Questionnaire: Patient

7.1)	Considerate of other people's feelings	Not True	Certainly True	n=23	av.=2,7	md=3	dev.=0,6
7.2)	Restless, overactive, cannot stay still for long	Not True	Certainly True	n=24	av.=1,6	md=1	dev.=0,8
7.3)	Often complains of headaches, stomach-aches or sickness	Not True	Certainly True	n=24	av.=1,6	md=1	dev.=0,8
7.4)	Shares readily with other children, for example toys, treats, pencils	Not True	Certainly True	n=24	av.=2,4	md=3	dev.=0,7
7.5)	Often loses temper	Not True	Certainly True	n=24	av.=2	md=2	dev.=0,8
7.6)	Rather solitary, prefers to play alone	Not True	Certainly True	n=24	av.=1,9	md=2	dev.=0,9
7.7)	Generally well behaved, usually does what adults request	Not True	Certainly True	n=24	av.=2,2	md=2	dev.=0,8
7.8)	Many worries or often seems worried	Not True	Certainly True	n=23	av.=2	md=2	dev.=0,9
7.9)	Good attention span, sees chores or homework through to the end	Not True	Certainly True	n=23	av.=2,3	md=2	dev.=0,7

9. CASP Sibling

9.1)	Social, play or leisure activities with family members at home (e.g., games, hobbies, "hanging out")	Age Expected	Not applicable	n=15	av.=1,4	md=1	dev.=1,1
9.2)	Social, play or leisure activities with friends at home (can include conversations on the phone or internet)	Age Expected	Not applicable	n=15	av.=1,3	md=1	dev.=1
9.3)	Family chores, responsibilities and decisions at home (For younger children this may be getting things or putting things away when asked or	Age Expected	Not applicable	n=15	av.=1,5	md=1	dev.=1,1
9.4)	Self-care activities (e.g., eating, dressing, bathing, combing or brushing hair, using the toilet)	Age Expected	Not applicable	n=15	av.=1,4	md=1	dev.=1,1
9.5)	Moving in and around the home	Age Expected	Not applicable	n=15	av.=1,4	md=1	dev.=1,1
9.6)	Communicating with other children and adults at home	Age Expected	Not applicable	n=15	av.=1,4	md=1	dev.=1,1
9.7)	Social, play, or leisure activities with friends in the neighborhood and community (e.g., casual games, “hanging out,” going to public places	Age Expected	Not applicable	n=15	av.=1,4	md=1	dev.=1,1
9.8)	Structured events and activities in the neighborhood and community (e.g., team sports, clubs, holiday or religious events,	Age Expected	Not applicable	n=15	av.=1,7	md=1	dev.=1,4
9.9)	Moving around the neighborhood and community (e.g., public buildings, parks, restaurants, movies) [Please consider your	Age Expected	Not applicable	n=15	av.=1,7	md=1	dev.=1,4
9.10)	Communicating with other children and adults in the neighborhood and community	Age Expected	Not applicable	n=15	av.=1,6	md=1	dev.=1,4
9.11)	Educational (academic) activities with other children in his or her classroom at school	Age expected	Not applicable	n=15	av.=1,6	md=1	dev.=1,4
9.12)	Social, play and recreational activities with other children at school (e.g., “hanging out,” sports, clubs, hobbies, creative arts, lunchtime	Age expected	Not applicable	n=15	av.=1,7	md=1	dev.=1,4

Item	Left pole	Right pole	n	av.	md	dev.
9.13) Moving around at school (e.g., to get to and use bathroom, playground, cafeteria, library or other rooms and things that are available to	Age expected	Not applicable	n=15	av.=1,6	md=1	dev.=1,4
9.14) Using educational materials and equipment that are available to other children in his or her classroom/s or that have been modified for	Age expected	Not applicable	n=15	av.=1,6	md=1	dev.=1,4
9.15) Communicating with other children and adults at school	Age expected	Not applicable	n=15	av.=1,6	md=1	dev.=1,4
9.16) Household activities (e.g., preparing some meals, doing laundry, washing dishes)	Age expected	Not applicable	n=15	av.=1,9	md=1	dev.=1,5
9.17) Shopping and managing money (e.g., shopping at stores, figuring out correct change)	Age expected	Not applicable	n=15	av.=1,7	md=1	dev.=1,4
9.18) Managing daily schedule (e.g., doing and completing daily activities on time; organizing and adjusting time and schedule when needed)	Age expected	Not applicable	n=15	av.=1,7	md=1	dev.=1,4
9.19) Using transportation to get around in the community (e.g., to and from school, work, social or leisure activities) [Driving vehicle or	Age expected	Not applicable	n=15	av.=1,7	md=1	dev.=1,4
9.20) Work activities and responsibilities (e.g., completion of work tasks, punctuality, attendance and getting along with supervisors	Age expected	Not applicable	n=15	av.=1,7	md=1	dev.=1,4

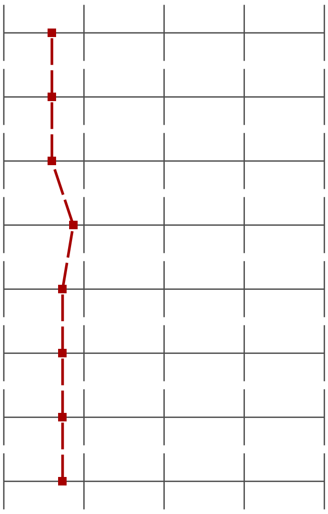

10. SD-Questionnaire Sibling

Item	Left pole	Right pole	n	av.	md	dev.
10.1) Considerate of other people's feelings	Not True	Certainly True	n=16	av.=2,6	md=3	dev.=0,7
10.2) Restless, overactive, cannot stay still for long	Not True	Certainly True	n=16	av.=1,4	md=1	dev.=0,5
10.3) Often complains of headaches, stomach-aches or sickness	Not True	Certainly True	n=16	av.=1,4	md=1	dev.=0,6
10.4) Shares readily with other children, for example toys, treats, pencils	Not True	Certainly True	n=16	av.=2,4	md=3	dev.=0,7
10.5) Often loses temper	Not True	Certainly True	n=16	av.=1,3	md=1	dev.=0,6
10.6) Rather solitary, prefers to play alone	Not True	Certainly True	n=16	av.=1,4	md=1	dev.=0,6
10.7) Generally well behaved, usually does what adults request	Not True	Certainly True	n=16	av.=2,3	md=2,5	dev.=0,8
10.8) Many worries or often seems worried	Not True	Certainly True	n=16	av.=1,5	md=1	dev.=0,7
10.9) Good attention span, sees chores or homework through to the end	Not True	Certainly True	n=16	av.=2,4	md=2,5	dev.=0,7

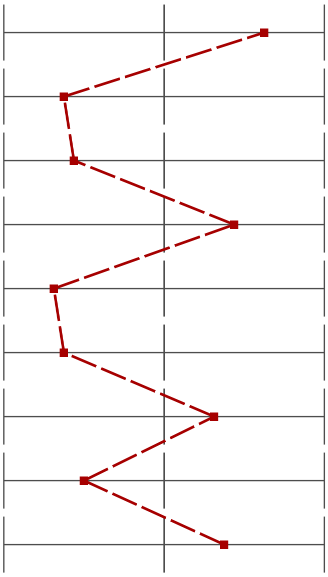

Comments Report

1. General aspects of pACC treatment

1.1) In which country is/ was your child being treated?

- Algeria (2 Counts)
- algerie
- Brasil (2 Counts)
- Deutsch
- Deutschland (5 Counts)
- España (2 Counts)
- Italia
- Italy (3 Counts)
- Poland
- Polska
- Slovakia
- Turkey (2 Counts)
- Türkiye (2 Counts)

1.2) What was the age of your child at diagnosis of adrenal cortical tumor? (in years and if applicable in months)

- 2
- 2 Jahre 8 Monate
- 3
- 4 Jahre 4 Monate
- 4 lata i 1 miesiąc
- 5
- 5 anos
- 5 ans et 07 mois
- 06
- 6 Jahre 5 Monate
- 6 weeks
- 7 jahre 5 Monate
- 7años y 9 meses
- 10 años y 7 meses
- 10.5
- 12
- 15 Jahre und 6 Monate
- 15 years
- 16 Jahre
- 16.5

- 17 months
- 18 Jahre alt
- 19 dias de vida
- 26 months

[1.3)] In which year was the tumor diagnosed? (e.g. 2010)

- 1995
- 2008
- 2009
- 2013
- 2016
- 2019
- 2020
- 2021 (4 Counts)
- 2022 (3 Counts)
- 2023 (6 Counts)
- 2024 (4 Counts)

[1.8)] What was the approx. weight and height of your child at diagnosis?

- 0 kg 0 cm
- 6 kg cm
- 6,5 kg 58 cm
- 11 kg cm
- 11, kg 81 cm
- 13 kg 96 cm
- 14 kg 90 cm
- 19 kg 88 cm
- 19 kg 108 cm
- 23 kg 118 cm (2 Counts)
- 23 kg 128 cm
- 24 kg 132 cm
- 29 kg 119 cm
- 31 kg 114 cm
- 39 kg 145 cm
- 40 kg 145 cm
- 52 kg 165 cm
- 57 kg 167 cm
- 67 kg 150 cm
- 74 kg 179 cm
- 80 kg 160 cm

- 110 kg   176 cm

1.9) What is the approx. weight and height of your child at the moment?

- 10 kg 78 cm
- 13, kg 98 cm
- 14, kg 98 cm
- 17 kg 113 cm
- 18 kg 100 cm
- 20 kg 118 cm
- 24 kg 115 cm
- 25 kg 130 cm
- 26 kg 135 cm
- 27 kg 134 cm
- 28 kg 123 cm
- 35 kg 135 cm
- 35 kg 147 cm
- 50 kg 150 cm
- 58 kg 162 cm
- 60 kg 172 cm
- 61 kg 171 cm
- 65 kg 157 cm
- 74 kg 180 cm
- 79 kg 182 cm
- 82 kg 162 cm
- 84 kg 178 cm
- 85 kg 181 cm
- 110 kg 176 cm

2. General aspects of mitotane treatment

2.1) What is/ was the duration of mitotane intake? (in months or years)

- 1 ano
- 1 Jahr
- 1 years
- 2 anni e mezzo
- 2 Jahre
- 2 Jahre, gewünschter Spiegel nie erreicht
- 2 years
- 3 Jahre 10 Monate
- 3 months
- 3 years

- 4 months
- 5 meses
- 09 month
- 10 months
- 11 miesięcy
- 12 mesi
- 14 Monate
- 19 months
- 20 Monate
- 24 months
- Casi 11 meses
- décembre 2024
- Mitotane start: 29.08.2008
  Mitotane stop: 27.02.2012
  total lenght of therapy:3 years and 6 months
- Un año y medio con interrupciones por intoxicación

2.3) If not, in which year was mitotane treatment ended up?

- 8.23
- 9.23
- 1998
- 2011
- 2012
- 2014
- 2019
- 2020
- 2022
- 2024 (2 Counts)

3. Adverse effects of mitotane treatment

3.2) If yes, please list the adverse effects here:

- Afectación neurológica elevada, cansancio físico y náuseas
- Alteração na linguagem
  Aréola escura
- Darmverschluss, Gedächtnisstörung, Zitrichkeit
- disturbi neurologici
  pubertà precoce
- drżenie rąk, zaburzenia równowagi, rozchwianie emocjonalne, zaburzenia koordynacji ruchowej, osłabienie siły mięśniowej, problemy z chodzeniem, zaburzenia koncentracji, kłopoty z pamięcią, problemy z mową, opóźniony rozwój psychomotoryczny, bóle brzucha, niewielkie powiększenie piersi i pojawienie się lekkiego owłosienia łonowego
- Fatique, Drowsiness
- ginecomastia
  insufficienza surrenalica
  ipotiroidismo

- Gleichgewichtsstörungen, Konzentrationsstörungen, Übelkeit, Bauchschmerzen, Kopfschmerzen, sehr wackelig auf den Beinen, teilweise nächtliches einnässen wenn der Spiegel zu hoch war

- Gleichgewichtsstörungen, Pupertas präcox

- il ce nerve

- Loss of appetite, 'enlarged' breasts, delay in speech according to the parents

- Medikament zu groß und Schluck Schwierigkeiten

- Mitotane given orally dissolved in glucose was irritating to pharynx, esophagus- caused problems with eating, vomiting and hypoglycemia and
  failure to thrive
  Mother noticed hypotonia, developmental delay, problems with balance, delayed walking, delayed small and big motoric development- problems with grasping things with hands, he couldnt eat with spoon
  he was feeding by syringe for over 4 years- he could eat only mixed things for over 4 years
  he had also tonic clonic seizures very frequently on
  Mitotane caused problems with veins, he needed flebotomy or blood was taken from bone/ or drugs during tonic clonic seizures was given to the bone. Tonic clonic seizures were caused by HYPOGLYCEMIA not by epilepsy.

- neurologic effect
  precoce puberty
  adrenal insufficiency

- Náuseas, vómitos, dolor de tripa, estómago revuelto, perdida de apetito, falta de equilibrio

- pubertà precoce

- Sonolência
  Crises a drenais
  Perda de peso
  Falta de apetite
  Cansaço
  Choro

- Sprachfindungsstörungen, Gedächtnisstörung, Konzentrationsstörungen, Brustwarzenschwellung, Gleichgewichtsstörung, körperliche Ausdauer stark eingeschränkt (schnell erschöpft), Ausschlag an den Ellenbögen, Vergesslichkeit (Verwirrung)

- Stomach burning, aggressiveness

- Tremors of the hands

- Weight loss, fatigue, loss of appetite

- yes

4. Medical history

4.2) If you chose "other" pre-existing conditions at the previous question, please list here:

- Asthma Bronchiale

- Beckwith-Wiedemann-Syndrom

  Hyperinsulinismus bis zum 14. Lebensjahr

- Bluthochdruck, verhäuft Kopfschmerzen und Bauchschmerzen

- Factor V Leyden

- Hoje está apenas em acompanhamento

4.4) If you chose others, please describe here:

- (according to the parents)

- 1. sweating but he is also overweight
  2. he is weak more than parents
  3. he is weaker than his brother

6. Child and Adolescent Scale of Participation (CASP): Patient

6.21) Please describe the type of things that interfere with your child's participation in the above-mentioned activities (e.g., things that your child does or that others do; or things about your home, school or community):

- Das Verständnis ist Entwicklungsverzögert seit der Mitotane Therapie, wird aber ganz langsam besser
- Dolores que no le permiten hacer ciertas cosas
- Dziecko jest nieodpieluchowane w nocy-brak umiejętności kontrolowania mikcji podczas snu. Mała motoryka (pisanie, rysowanie) poniżej oczekiwań względem wieku.
- Ele faz tudo com bis capacidade e desempenho
- Ele é um bebê.
- Keine festen Freunde, geringe Frustrationstoleranz, Grenzen von anderen beachten
- Körperliche Einschränkung beim Sport, Ausdauer
- Mein Kind ist in seiner Konzentration und körperlichen und geistigen Ausdauer stark eingeschränkt. Sein Sprachvermögen ist eingeschränkt und schwankend. Er ist auf eine Schulbegleitung angewiesen.
- none
- Physical appearance, surgery related factors

6.22) Please describe the type of things that help with your child's participation in the above-mentioned activities (e.g., things that your child does or that others do; or things about your home, school or community):

- Integrationskraft
- Kein Besuch auf Großveranstaltungen oder Übernachtungen zwecks Medikamenteneinnahme
- No tener dolor
- Okazjonalna pomoc w nauce pisania i rysowania, ćwiczenia praktyczne małej motoryki.
- Participa de tudo com normalidade
- Schulbegleitung, Logopädie......
- verbal encouragement by parents
- We try to perform different activities at home
- Wir begleiten unsere Tochter bei allen Aktivitäten, das gibt ihr Sicherheit und sie traut sich immer mehr

6.24) If yes, please identify:

The evaluation will not be displayed due to low response rate.

6.26) If yes, please identify:

- During treatment period, separate room, separate furnitures etc
- El año pasado tuvo alguna pequeña adaptación curricular
- The table and the chairs have been rearranged due to flatulence/bloating after heavy Meal

8. Questionnaire for the patient's sibling

8.1) Does the sibling (closest in age to the patient and used for the tests in addition) have any pre-existing conditions, e.g. development delays? If yes, please list here:

- Asthma Bronchiale
- Bem saudável.
- Her older brother has a diagnosis of Joubert syndrome, he has epilepsy, developmental delay
- Mein Kind hat keine Geschwister

- Nein (2 Counts)
- no (3 Counts)
- No
- Não há atraso no desenvolvimento

9. CASP Sibling

9.21) Please describe the type of things that interfere with your child’s participation in the above-mentioned activities (e.g., things that your child does or that others do; or things about your home, school or community):

- Bebê
- Because he has delays in many aspects he can not do everything his peers can do easily.
- Die kleine Schwester ist sehr zuvorkommend und möchte immer jedem helfen
- Dolores en alguna parte del cuerpo
- Nada interfere, participa em tudo normalmente

9.22) Please describe the type of things that help with your child’s participation in the above-mentioned activities (e.g., things that your child does or that others do; or things about your home, school or community):

- No tener dolor
- Participa em tudo normalmente

9.24) If yes, please identify:

The evaluation will not be displayed due to low response rate.

9.26) If yes, please identify:

- Rearranging furniture
