# Supplementary material for: Functional and behavioral outcomes in pediatric adrenal carcinoma under mitotane therapy: a caregiver-reported pilot study
Source: Endocr Oncol. 2026 Jun 5;6(1):e260010. doi: 10.1530/EO-26-0010 (PMC13250649; doi:10.1530/EO-26-0010)
Supplement: Supplementary file 2 [file supplementary_tables.pdf]

Table S 1: Development of age- and sex-standardized length (in cm) and BMI (in kg/m²) between point of diagnosis and point of survey; age in months (= mo); F = Female; M = Male

		**Point of Diagnosis**					**Point of Survey**						
**ID**	**Sex**	**Age** mo	**Length** cm	**Length Z score**[a]	**BMI** kg/m^2	**BMI Z Score**[a]	**Age** mo	**Length** cm	**Length Z score**[a]	**BMI** kg/m^2	**BMI Z Score**[a]	**ΔZ Length**[d]	**ΔZ BMI**[g]
1	F	32	NA	NA	NA	NA	80	113	-1.11	13.3	-1.48	NA	NA
2	F	24	88	0.71	24.5	4.74	84	123	0.40	18.5	1.52	-0.31	-3.22
3	F	60	118	1.80	16.5	0.77	228	162	-0.18	22.1	0.21	-1.98	-0.56
4	M	36	90	-1.64	17.3	1.28	72	100	-3.24	18	1.72	-1.60	0.44
5	F	216	176	1.96	35.5	2.90	312	176	NA	35.5	NA	NA	NA
6	M	144	145	-0.58	19	0.65	168	147	-2.10	16.2	-1.50	-1.53	-2.14
7	F	72	118	0.56	16.5	0.73	84	130	1.68	14.8	-0.38	1.12	-1.11
8	M	1.5	58	0.90	19.3	2.45	193	178	0.63	26.5	1.71	-0.27	-0.74
9	M	77	132	2.68	13.8	-1.29	89	135	2.01	14.3	-0.98	-0.67	0.31
10	F	52	119	3.13	20.5	2.90	88	135	2.20	19.2	1.72	-0.92	-1.18
11	M	89	NA	NA	NA	NA	221	180	0.49	22.8	0.30	NA	NA
12	F	127	145	0.41	18.5	0.65	163	150	-1.23	22.2	0.96	-1.64	0.31
13	M	49	108	0.97	16.3	0.74	73	118	0.31	14.4	-0.73	-0.66	-1.47
14	M	186	179	1.01	23.1	3.18	222	182	0.76	23.8	0.60	-0.25	-2.58
15	M	93	128	0.38	14	-1.30	105	134	0.46	15	-0.66	-0.90	0.64
16	M	126	165	3.76	19.1	1.11	150	172	2.69	20.3	0.97	-1.06	-0.14
17	F	198	150	-1.89	29.8	2.12	210	157	-0.90	26.4	1.41	0.99	-0.71
18	M	60	NA	NA	NA	NA	408	181	NA	25.9	NA	NA	NA
19	M	1	NA	NA	NA	NA	13	78	0.45	16.4	-0.21	NA	NA
20	M	67	114	0.12	23.9	4.34	67	115	0.33	18.1	1.85	0.21	-2.49
21	M	180	167	-0.25	20.4	0.25	192	171	-0.24	20.9	0.15	0.01	-0.09
22	M	26	96	2.27	14.1	-1.63	38	98	0.16	13.5	-1.80	-2.10	-0.18
23	F	17	81	0.47	16.8	0.69	41	98	-0.10	14.6	-0.56	-0.57	-1.25
24	F	192	160	-0.37	31.3	2.39	204	162	-0.13	31.2	2.32	0.24	-0.07
**Mean ± SD**				0.82 ± 1.47[b]		1.38 ± 1.75[e]			0.15 ± 1.36[c]		0.33 ± 1.24[f]	-0.57± 0.93	-1.06± 1.12

[a]*Z score Calculation:* $Z=((X/M)^{L}-1)/(L \times S)$ *with X=BMI or Length and L, M, S from WHO Reference Values*

[b]*Length Z score at Diagnose: n = 20, Mean ± SD = 0.82 ± 1.47; Min/Max =-1.89/3.76;*

[c]*Length Z score at Survey: n = 22; Mean ± SD = 0.15± 1.36; Min/Max = -3.24/2.69;*

[d]*Comparison of length Z-Score at diagnosis and at survey: Paired t-test: t (18) = 2.708, p = 0.014*, 95% CI = [-1.022, -0.129],* $R^2 = 0.29$.

[e]*BMI-Z-Score at Diagnose: n = 20, Mean ± SD = 1.38 ± 1.75; Min/Max = -1.63/4.74;*

[f]*BMI-Z-Score at Survey: n = 22; Mean ± SD = 0.33 ± 1.24; Min/Max = -1.80/2.32;*

[g]*Comparison of BMI-Z-Score at diagnosis and at survey: Paired t-test: t (18) = 3.33, p = 0.004**, 95% CI = [-1.394, -0.315],* $R^2 = 0.38$.

Table S 2: CASP score calculation for the entire cohort and the subgroups with ongoing mitotane treatment, completed mitotane treatment, and siblings per domain; n = number of responses, SD = Standard deviation

		**CASP score**			
**Domain**		all Patients	Mitotane ongoing	Mitotane completed	Siblings
Home participation	n	23	11	12	14
	median	95.8	87.5	97.9	100.0
	mean	89.1	82.6	95.1	96.1
	SD	14.3	17.1	7.9	7.8
Community participation	n	22	10	12	14
	median	96.9	75.0	100.0	100.0
	mean	86.4	79.4	92.2	96.0
	SD	17.2	19.1	13.6	9.0
School participation	n	17	6	11	13
	median	95.0	77.5	100.0	100.0
	mean	89.9	77.3	96.8	97.7
	SD	13.6	15.5	5.1	7.0
Home & Community living	n	20	9	11	13
	median	95.0	90.0	100.0	100.0
	mean	90.9	89.3	92.3	93.5
	SD	4.3	15.5	13.9	11.4
Overall CASP Score	n	23	11	12	14
	median	91.3	86.7	98.3	100.0
	mean	88.1	81.5	94.2	96.0
	SD	13.8	15.3	12.0	8.2

Table S 3: CASP scores divided into four categories (full participation, somewhat limited, limited, very limited) per domain and per group (entire cohort, ongoing mitotane, completed mitotane, siblings); NA = not applicable

**Domain**	**Category**[1]	**all Patients** (n = 24)		**Mitotane ongoing** (n = 12)		**Mitotane completed** (n = 12)		**Siblings** (n = 17)	
		n	%[2]	n	%[2]	n	%[2]	n	%[2]
Home participation	1	8	34.8	2	18.2	6	50.0	10	71.4
	2	10	43.5	5	45.5	5	41.7	3	21.4
	3	3	13.0	2	18.2	1	8.3	1	7.1
	4	2	8.7	2	18.2	0	0.0	0	0.0
	NA	1	-	1	-	0	-	3	-
Community participation	1	11	50.0	3	30.0	8	66.7	11	78.6
	2	4	18.2	1	10.0	3	25.0	2	14.3
	3	4	18.2	4	40.0	0	0.0	1	7.1
	4	3	13.6	2	20.0	1	8.3	0	0.0
	NA	2	-	2	-	0	-	3	-
School participation	1	7	41.2	0	0.0	7	63.6	11	84.6
	2	6	35.3	2	33.3	4	36.4	1	7.7
	3	3	17.6	3	50.0	0	0.0	1	7.7
	4	1	5.9	1	16.7	0	0.0	0	0.0
	NA	7	-	6	-	1	-	4	-
Home & Community living	1	9	45.0	3	33.3	6	54.5	9	69.2
	2	8	40.0	5	55.6	3	27.3	1	7.7
	3	1	5.0	0	0.0	1	9.1	2	15.4
	4	2	10.0	1	11.1	1	9.1	1	7.7
	NA	4	-	3	-	1	-	4	-
Overall CASP Score	1	7	30.4	1	9.1	6	50.0	9	64.3
	2	11	47.8	6	54.5	5	41.7	3	21.4
	3	3	13.0	2	18.2	1	8.3	1	7.1
	4	2	8.7	2	18.2	0	0.0	0	0.0
	NA	1	-	1	-	0	-	3	-

[1]*Category 1: CASP score 100–97.5 = Full participation; Category 2: CASP score 97.5–81.0 = Somewhat limited participation; Category 3: CASP score 81.0–68.5 = Limited participation; Category 4: CASP score 68.5 or less = Very limited participation*

[2]*Percentages are calculated within each domain considering only valid responses (NAs excluded)*

Table S 4: Answers from the SD-Questionnaire for the entire cohort and the subgroups with ongoing mitotane treatment, completed mitotane treatment, and siblings; n = number of responses, SD = Standard deviation

		**SD-Questionnaire answer**			
**Item**		all Patients	Mitotane ongoing	Mitotane completed	Siblings
Often complains of headaches, stomach-aches or sickness	n	24	12	12	16
	median	1.00	2.00	1.00	1.00
	mean	1.63	1.83	1.42	1.44
	SD	0.77	0.72	0.79	0.63
Many worries or often seems worried	n	23	11	12	16
	median	2.00	2.00	2.00	1.00
	mean	2.00	2.09	1.92	1.50
	SD	0.85	0.83	0.90	0.73
Often loses temper	n	24	12	12	16
	median	2.00	2.50	1.00	1.00
	mean	2.00	2.42	1.58	1.31
	SD	0.83	0.67	0.79	0.60
Generally well behaved, usually does what adults request	n	24	12	12	16
	median	2.00	2.00	3.00	2.50
	mean	2.21	2.00	2.42	2.31
	SD	0.78	0.74	0.79	0.79
Restless, overactive, cannot stay still for long	n	24	12	12	16
	median	1.00	2.00	1.00	1.00
	mean	1.58	2.00	1.17	1.38
	SD	0.83	0.85	0.58	0.50
Good attention span, sees chores or homework through to the end	n	23	11	12	16
	median	2.00	2.00	3.00	2.50
	mean	2.30	2.09	2.50	2.38
	SD	0.70	0.70	0.67	0.72
Rather solitary, prefers to play alone	n	24	12	12	16
	median	2.00	2.50	1.00	1.00
	mean	1.88	2.25	1.50	1.38
	SD	0.90	0.87	0.80	0.62
Considerate of other people's feelings	n	23	11	12	16
	median	3.00	3.00	3.00	3.00
	mean	2.70	2.45	2.92	2.63
	SD	0.56	0.69	0.29	0.72
Shares readily with other children, for example toys, treats, pencils	n	24	12	12	16
	median	3.00	2.00	3.00	3.00
	mean	2.42	2.08	2.75	2.44
	SD	0.72	0.79	0.45	0.73
